# Positron emission tomography neuroimaging of [^18^F]fluorodeoxyglucose uptake and related behavior in the *Pink1*−/− rat model of Parkinson disease

**DOI:** 10.3389/fnins.2024.1451118

**Published:** 2024-10-15

**Authors:** Alexander K. Converse, Maryann N. Krasko, Denis Michael Rudisch, Charlie Lenell Lunaris, Alex F. Nisbet, Maxim S. Slesarev, John C. Szot, Andrew G. Hoerst, Glen E. Leverson, Catherine L. Gallagher, Michelle R. Ciucci

**Affiliations:** ^1^Waisman Center, University of Wisconsin-Madison, Madison, WI, United States; ^2^Division of Otolaryngology, Department of Surgery, University of Wisconsin-Madison, Madison, WI, United States; ^3^Department of Communication Science and Disorders, University of Wisconsin-Madison, Madison, WI, United States; ^4^Institute for Clinical and Translational Research, University of Wisconsin-Madison, Madison, WI, United States; ^5^Department of Neurology, University of Wisconsin-Madison, Madison, WI, United States; ^6^Neuroscience Training Program, University of Wisconsin-Madison, Madison, WI, United States

**Keywords:** Parkinson disease, *Pink1−/−* rat model, positron emission tomography, [^18^F]fluorodeoxyglucose, ultrasonic vocalizations, Five-Choice Serial Reaction Time Task, Tapered Balance Beam, Cylinder Test

## Abstract

**Introduction:**

Parkinson disease (PD) is a neurodegenerative condition affecting multiple sensorimotor and cognitive systems. The *Pink1−/−* rat model exhibits vocal, cognitive, and limb use deficits seen in idiopathic PD. We sought to measure glucose metabolism in brain regions in *Pink1−/−* and wild type (WT) rats, and to associate these to measures of ultrasonic vocalization, cognition, and limb use behavior.

**Methods:**

*Pink1−/−* (n = 12) and WT (n = 14) rats were imaged by [^18^F]fluorodeoxyglucose (FDG) positron emission tomography (PET) in a repeated measures design at approximately 10 months of age and 6 weeks later. Relative regional glucose metabolism was indexed by whole brain normalized FDG uptake, which was calculated for 18 regions identified *a priori* for comparison. Behavioral measures included tests of communication via ultrasonic vocalization, cognition with 5-Choice Serial Reaction Time Test (5-CSRTT), and limb use with Cylinder Test and Challenge Beam.

**Results:**

Relative glucose metabolism was significantly different in *Pink1−/−* rats in prelimbic area, striatum, nucleus ambiguus, globus pallidus, and posterior parietal association cortex compared to WT controls. For behavioral measures, *Pink1*−/− rats demonstrated quieter vocalizations with a restricted frequency range, and they showed increased number of foot-faults and hindlimb steps (shuffling) in limb motor tests. Significant behavior vs. brain correlations included associations of ultrasonic vocalization parameters with glucose metabolism indices in locus coeruleus and substantia nigra.

**Conclusion:**

FDG PET reveals abnormalities in relative regional brain glucose metabolism in *Pink1−/−* rats in brain regions that are important to cognition, vocalization, and limb motor control that are also impacted by Parkinson disease. This method may be useful for mechanistic studies of behavioral deficits and therapeutic interventions in translational studies in the *Pink1−/−* PD model.

## 1 Introduction

Parkinson disease (PD) is a progressive neurodegenerative disorder that manifests throughout the central nervous system. In addition to typical motor dysfunction (i.e., gait disturbances, tremor) (Berg et al., 2018; Bloem et al., 2021), PD results in other motor and nonmotor deficits including difficulties with speech (Logemann et al., 1978; Hartelius and Svensson, 1994; Theodoros, 2011), swallowing (Kalf et al., 2012; Suttrup and Warnecke, 2016; Schapira et al., 2017; Pflug et al., 2018; Krasko et al., 2023a; Rudisch et al., 2023b), and cognition (Aarsland et al., 2017; O’Callaghan and Lewis, 2017). The role of nigrostriatal dopamine in motor functioning is widely known. Its depletion is well-understood to lead to motor decline in PD and this premise has guided therapeutic dopamine replacement as treatment for PD. However, many other sensorimotor and nonmotor symptoms of PD appear in the prodromal stage of disease, prior to significant nigrostriatal depletion (Schapira et al., 2017). Moreover, dopamine-centered treatments do not improve vocal communication, swallowing, or cognitive deficits (Ho et al., 2008; Skodda et al., 2010; Ciucci et al., 2013; Seppi et al., 2019). Currently, the mechanisms driving many of these other common PD deficits are not well understood.

Glucose metabolism is associated with the pathophysiology of PD (Dunn et al., 2014; Dai et al., 2023). Glucose serves as the primary energy source for the brain, and disruptions in its metabolism can lead to cellular energy deficits and oxidative stress, both of which are implicated in neurodegeneration (Anandhan et al., 2017). Dysregulated glucose metabolism can also exacerbate mitochondrial dysfunction (Anandhan et al., 2017), impair protein degradation, and promote neuroinflammation (Edison et al., 2013), all of which are key pathological features of PD. Clinically, impaired glucose utilization has been observed in the brains of individuals with PD through positron emission tomography (PET) imaging using the PET tracer [^18^F]fluorodeoxyglucose (FDG), a glucose analog (Teune et al., 2014). A pattern of resting state metabolism has been identified as hypermetabolic in several brain regions including the thalamus (Thal), putamen, globus pallidus, pons, cerebellum and motor cortex, as well as hypometabolic in posterior parietal, occipital, and frontal cortices (Eidelberg, 2009; Meles et al., 2020). The exact brain-behavior relationships between vocal communication/cognition and glucose metabolism in associated brain regions in the PD disease process are yet to be uncovered. Elucidating these relationships is imperative for better understanding the pathogenesis of PD, as well as the development of novel, targeted therapeutics for these specific PD deficits.

Repeated *in vivo* imaging enhances mechanistic understanding of potential treatments, allowing for within subject control that increases statistical power, and it further permits the association of baseline measures with subsequent responses (i.e., potential neuroprogression over time) (Nikolaus et al., 2004; Lancelot and Zimmer, 2010). Animal models offer a controlled environment where variables can be manipulated with greater precision, facilitating more rigorous assessment of glucose uptake and behavior within the same organism over time (Konnova and Swanberg, 2018). The *Pink1−/−* rat model is based on the disruption of the Pink1 gene, which encodes PTEN-induced putative kinase 1 (PINK1), a protein associated with mitochondrial function and implicated in the development of PD (Quinn et al., 2020). In *Pink1−/−* rats, the absence of PINK1 leads to mitochondrial dysfunction, oxidative stress, and impaired cellular energy metabolism, characteristics observed in human PD (Quinn et al., 2020). Moreover, this is a progressive model of PD, where deficits manifest and worsen as the rat ages and disease progresses (Dave et al., 2014; Grant et al., 2015). Specifically, the *Pink1−/−* rat demonstrates early and progressive decline in swallowing/oromotor function (Krasko et al., 2023b), anxiety (Hoffmeister et al., 2021, 2022), affect (Marquis et al., 2020), as well as vocal communication (Grant et al., 2015; Johnson et al., 2020; Marquis et al., 2020; Krasko et al., 2021), cognition (Soto et al., 2024), and limb motor function (Dave et al., 2014; Grant et al., 2015; Kelm-Nelson et al., 2018), making it an appropriate model for this work. Additionally, this model demonstrates loss of dopaminergic cells in the substantia nigra (SN) (Dave et al., 2014), changes in norepinephrine levels in SN and locus coeruleus (LC) (Grant et al., 2015; Kelm-Nelson et al., 2018), and correlations between brainstem noradrenergic markers and vocal decline/anxiety (e.g., β_1_ receptor in the nucleus ambiguus/dorsal motor nucleus of vagus and reduced vocal intensity) (Hoffmeister et al., 2021). Animal models of PD have been imaged by PET, and *Pink1−/−* rats have been studied with MRI (Ferris et al., 2018; Real et al., 2023), however, to our knowledge, the *Pink1−/−* model has not yet been imaged by PET. We expect that these methods and the use of more specific PET radiotracers in the *Pink1−/−* model may lead to improved understanding of the relations among brain and behavior and eventually aid in translation of interventions to the clinic.

Here we describe the novel application of FDG PET neuroimaging in the *Pink1−/−* genetic rat model of PD and associations with behavioral measures. With this application of PET neuroimaging in *Pink1−/−* rats, we sought to establish methods for future studies and to test the hypotheses that *Pink1−/−* rats would show, in comparison to wild type (WT) controls, (1) altered relative regional brain glucose metabolism (i.e., FDG uptake), (2) diminished behavioral function, (3) altered associations between behavioral measures and metabolism, and (4) more rapid degeneration. Given our overarching interest in catecholamines in the *Pink1−/−* model, in the FDG PET analysis we focused on brain regions that are key components of the noradrenergic and dopaminergic systems. In particular, we examined regions rich in norepinephrine transporters, including LC, Thal, and prelimbic area (PrL), as well as regions with high densities of dopamine transporters, including SN, striatum (Str), and PrL. Furthermore, we examined regions implicated in neuromotor speech difficulties (dysarthria), namely hypoglossal nucleus, solitary nucleus, nucleus ambiguus, and periaqueductal grey. Additionally, we assessed regions consistently found to be abnormal on FDG PET in PD, including globus pallidus, pons, primary and secondary motor areas, medial and posterior parietal association cortex areas, caudate putamen, cerebellum, and primary visual area.

## 2 Materials and methods

### 2.1 Study design

To test the hypotheses regarding genotype differences, brain-behavior associations, and age effects, rats were studied using a repeated measures design. As detailed below (Table 1), male *Pink1−/−* (*n* = 12) and male WT (*n* = 15) Long-Evans rats were assessed at baseline and then at a final timepoint 6 weeks later with no intervening treatment. A younger and older group of rats were chosen to span the age range from 9 to 12 months, a window in which our lab has observed declines in various measures in *Pink1−/−* rats. Rats underwent FDG PET brain imaging and behavioral assays, as described below. This manuscript was drafted in accord with the ARRIVE 2.0 guidelines (Percie du Sert et al., 2020).

**Table 1:** PET scans: mean ages and weights with SD.

Group	Genotype	Timepoint	n	Age (months)	Weight (g)
1	*Pink1−/−*	Baseline	7	9.15 ± 0.06	524 ± 48
		Final	8	10.74 ± 0.11	520 ± 48
	Wildtype	Baseline^a^	0		
		Final	7	10.44 ± 0.00	496 ± 34
2	*Pink1−/−*	Baseline	4	10.44 ± 0.00	578 ± 15
		Final	4	11.64 ± 0.00	547 ± 9
	Wildtype	Baseline	7	10.39 ± 0.06	526 ± 46
		Final	6	12.24 ± 0.00	548 ± 25

Mean ± s.d. Three additional scans were not included in the analysis because of poor IV injections of radiotracer.

a No WT baseline scans were obtained for the younger age group due to scanner malfunction.

### 2.2 Rats

All work involving rats was approved by the University of Wisconsin-Madison Institutional Animal Care and Use Committee (IACUC protocols M006390, M006782, and G006404) and was conducted in accordance with the National Institutes of Health Guide for the Care and Use of Laboratory Animals (2011). Twenty seven male Long-Evans rats [*Pink1−/−* (*n* = 12), WT (*n* = 15)] were received from Envigo Research Laboratories (Boyertown, PA, United States) at 4–6 weeks of age. Genotype was independently confirmed for rats used in this study (Transnetyx, Cordova, Tennessee). All handling and testing occurred during the dark period (awake) of a 12-h reverse light–dark cycle (12:12) under partial red illumination. Water and food were provided *ad libitum* except when used as a reward for the 5-Choice Serial Reaction Time Task (see below). Behavioral testing and *in vivo* imaging generally occurred twice for each rat at approximately 9–11 months of age with baseline and final testing separated by ~6 weeks. Rats were acclimated to handling and transport for 1 week prior to training and testing.

### 2.3 PET

#### 2.3.1 Data acquisition

Rats were imaged using a microPET P4 scanner (Concorde Microsystems Inc., Knoxville), with 7.8 cm axial and 19 cm transaxial fields of view, and 2 mm full width at half maximum resolution. Rats were scanned in groups of up to four. An in-house anesthesia system allowed for individual adjustment of isoflurane concentration in oxygen at 1 L/min. Anesthesia was initially delivered at 4.5% in an induction chamber, after which rats were placed in head holders affixed to the scanner bed. The holders were made in-house and consisted of two levels, each accommodating two rats with a tooth bar and ear bars for each individual rat to keep their heads stationary during the scans. Isoflurane at a concentration of 1–4% in oxygen at 1 L/min was administered individually in order to maintain anesthetic plane. Heating pads and mylar body wraps were used to keep the rats at an acceptable body temperature (35.9–37.5°C). Oxygen saturation, heart rate, and rectal temperature were monitored regularly, and ophthalmic lubricant was used to keep corneas moisturized throughout the procedure. Head holder, anesthesia, and warming apparatus materials within the field of view were minimized to reduce scatter and attenuation. 24-gauge intravenous (IV) catheters were placed in the tail veins and kept primed with heparinized saline (10 USP/mL). To dilate veins for IV catheter placement, tails were placed in 45°C water for 5 min and a tourniquet was placed at the base of the tail of each anesthetized rat at the end of the 5-min period. Catheter placement was checked by examining blood backflow, and intravenous administration was confirmed by examining time activity curves (TACs) in post-processing. Rats were centered axially at the interaural line. When scanned in groups of four, the rats were placed radially at 4.9 cm from the cFOV. When scanned in groups of two, radial placement was at 3.5 cm from cFOV. A ^57^Co point source was used to obtain a 518 s single-pass transmission scan with a 120–125 keV energy window. Once head placement was verified by examining the transmission image, a 90-min emission scan (350–650 keV with a 6 ns coincidence window) was started and a single bolus of FDG (Sofie Romeoville, Illinois) was injected into each rat at 1-min intervals (nominal 1 μCi/g, measured 0.58 ± 0.19 mCi net injected activity).

#### 2.3.2 Image reconstruction

Image reconstruction was performed using the scanner vendor’s software (microPET Manager and ASIPro, Siemens, Knoxville). List mode data were sorted into 90 1-min frames and binned into 3D sinograms (168 projection angles × 192 bins; span 3; ring difference 31; 11 segments). The emission sinograms were corrected for random coincidences, detector sensitivity, and dead time. Transmission sinograms were reconstructed, calibrated to a representative region of interest (ROI) in brain (511 keV attenuation coefficient μ = 0.095 cm^−1^), segmented (< 0.095/2 cm^−1^ = 0, > 0.095/2 cm^−1^ = 0.095 cm^−1^), and forward projected. Using the adjusted transmission sinograms to correct for attenuation and scatter, dynamic emission images were reconstructed by filtered back-projection (Fourier 2D rebinning; image matrix size, 128 × 128; pixel size, 0.47 mm × 0.47 mm in-plane × 1.21 mm slice thickness, ramp filter) and corrected for radioactive decay to the beginning of the scan.

#### 2.3.3 Image analysis

The publicly available Waxholm atlas of the rat brain was used for anatomical identification (version 1.01) (Papp et al., 2014). To facilitate the alignment procedure, the original 39 μm cubic voxels were downsampled to 0.25 mm cubic, which is still small compared to the 1.8 mm resolution of the PET scanner. This downsampling resulted in the loss of 2 small regions of the original 76. The remaining 74 bilateral regions had a median size of 6 mm^3^. The PrL ROI was obtained from version 4 of the Waxholm atlas (Kleven et al., 2023). Additional regions were delineated in-house with respect to the Paxinos atlas (Paxinos and Watson, 1998) for pons, nucleus ambiguus, LC, solitary nucleus, and hypoglossal nucleus (12). To judge if the Waxholm Sprague Dawley atlas was suitable for analysis of Long-Evans rats, publicly available Long-Evans MR images^1^ were aligned to the atlas MR template by nine degrees of freedom (df, 3 shifts, 3 rotations, and 3 scales). The resulting scaling in 3 dimensions increased the Long-Evans brain volume by 1.5% (scale factors: medial-lateral 1.011, dorsal-ventral 0.979, anterior–posterior 1.024), similar to a reported 4% smaller brain weight in LE at PND 35 (Holson et al., 2001). Waxholm atlas boundaries of the whole brain, cerebellum, LC, periaqueductal grey, Thal, Str, and PrL ROIs agreed to better than 0.5 mm with structures in the transformed Long-Evans MRI template.

Alignment of PET images to the atlas was performed using in-house python code that called freely available image processing algorithms (Jenkinson et al., 2002). Sum images were created from 30 to 60 min post-injection of radiotracer. PET images were first coarsely aligned by manual shifts and rotations (6 df) to the atlas space. The PET images were next finely aligned by computer optimization of 6 and then 9 df transforms to the brain mask, which was smoothed (Gaussian kernel, *σ* = 0.85 mm) to simulate the PET scanner resolution. For within-subject alignment, baseline and final timepoint image pairs of the same subject were first aligned to each other by 6 df and then with the same 9 df transformation to the Waxholm atlas. From these aligned images, baseline and final images of 6 WT and 6 *Pink1−/−* rats (24 images) were averaged and aligned by 9 df to the smoothed brain mask to create a study template. The fine alignment procedure was then repeated with the study template as the target. The final alignment transformations were created by concatenating the coarse and fine transformation matrices, and these were applied to the 4D images in scanner space yielding aligned dynamic images in atlas space.

To determine the regional distribution of FDG uptake, time-activity curves (TACs) were extracted for each atlas ROI. The mean radioactivity concentration (Bq/mL) from 30 to 60 min was calculated and divided by the injected dose per body weight (Bq/g) to yield the standardized uptake value (SUV, g/mL) for each region. To correct for any variations within or between rats in administration, distribution, metabolism, and excretion (ADME), each region’s SUV was divided by the whole brain SUV to yield the relative SUV (SUVr), which is equivalent to the regional radioactivity concentration scaled to the whole brain radioactivity concentration. SUVr served as an index of regional glucose metabolism relative to whole brain (Lin et al., 2008).

### 2.4 Ultrasonic vocalizations

Vocalization was tested using an established mating paradigm to elicit male rat ultrasonic vocalizations (Ciucci et al., 2010; Johnson et al., 2013; Hoffmeister et al., 2021; Broadfoot et al., 2023; Cullins et al., 2023; Rudisch et al., 2023a). Briefly, all testing rats were acclimated to the testing procedure for 7 consecutive days followed by one recording session. Testing occurred at baseline and final timepoints. Male testing rats were placed in their standard polycarbonate home cage without their housing-mate. An ultrasonic microphone with 16-bit resolution and a sampling rate of 250 kHz (CM16, Avisoft, Berlin, Germany) attached to an ultrasonic recording system (AviSoft, Berlin, Germany) was mounted 15 cm above the rat’s home cage. A stimulus female rat, conspecific in estrus, was placed in the single male rat’s cage and removed after two mounting attempts by the male rat or after the male rat had expressed interest (i.e., sniffing, genital auto-grooming, or chasing). Estrus of the stimulus female rats was confirmed by observing typical behavioral signs (e.g., lordosis, ear wiggling, hopping, and darting) (Bialy et al., 2000; Brudzynski, 2021). Ultrasonic vocalizations in the 50 kHz range were recorded for 5 min after the female rat was removed. USV files (WAV) were automatically detected and analyzed for frequency parameters [peak frequency and delta frequency (bandwidth)] in kHz, loudness [mean power (dB/Hz)], and call complexity parameters (% complex calls), using DeepSqueak v.3.0.1 (Coffey et al., 2019) in MATLAB (9.11.17.69968 [R2021b]; MathWorks Inc., Natick, MA). DeepSqueak identifies calls using a pre-trained neural network. USVs were then manually labeled for six call types based on the spectrogram output from DeepSqueak: simple, simple-compound, frequency-modulated, frequency-modulated-compound, harmonic, and harmonic-compound (Supplementary Table S1) and collapsed into three call categories (simple, frequency-modulated (FM), and harmonic) for statistical analysis (Wright et al., 2010; Hoffmeister et al., 2024). Peak frequency (kHz), mean power (dB/Hz), and bandwidth (kHz) of simple and frequency-modulated calls, parameters sensitive to abnormalities in social communication in rat models of PD (Krasko et al., 2021), were used for statistical analyses using baseline and final testing recordings.

### 2.5 Tapered Balance Beam

Motor performance and coordination were measured via Tapered Balance Beam as previously described (Grant et al., 2015). The Tapered Balance Beam and Cylinder Test (below) are two of the more common gross motor tests employed by our lab that are sensitive to sensorimotor changes in the *Pink1−/−* model at this age. Briefly, a beam (165 cm in length), with the testing length being the middle 135 cm, was suspended 1 m above the ground. The beam was wider on one end (6.35 cm), narrowing to 1.27 cm on the opposite end. The upper part of the beam is composed of high-density polyethylene plastic with a safety walk non-slip material on the top surface. Rats were acclimated to the task for three consecutive days prior to testing, completing five trials each day. On testing day, each rat was placed at the wide end of the beam and was video-recorded (Sony HDR-CX210, New York, NY) traversing the tapered beam to the narrower end for a total of five trials. Raters, blinded to age and genotype, viewed and rated recordings in slow motion using Windows Media Player (Microsoft, Redmond, WA). All personnel were trained to meet intra-and inter-rater reliability criteria of ICC >0.95. The total time to traverse the beam (sec), time to traverse the final one-third of the beam (sec), the number of combined forelimb foot faults, and the number of combined hindlimb foot faults along the length the beam were recorded for each trial.

### 2.6 Cylinder Test

Spontaneous motor activity was measured via Cylinder Test for 120 s as previously described (Grant et al., 2015). A transparent cylinder (20 cm diameter × 60 cm height) was used, which was placed vertically on a piece of glass. A camera (Sony HDR-CX210, New York, NY) was positioned below, in order to provide a clear view of movements along the ground and walls of the cylinder. Raters, blinded to age and genotype, viewed recordings in slow motion using Windows Media Player. All personnel were trained to meet intra-and inter-rater reliability criteria of ICC >0.95. The total number of forelimb up steps, the total number of forelimb down steps, the total number of hindlimb steps, the total number of rears, and the total number of lands were measured over a two-minute period for each rat.

### 2.7 Five-Choice Serial Reaction Time Task

Attention and impulse control were assessed with the 5-CSRTT using Noldus Ethovision XT software (Version 13.0.1220, Noldus Information Technology Inc., Leesburg, VA, United States) and a Med Associates five-choice chamber (Model MED-NP5L-B1, Med Associates Inc., St. Albans, VT, United States). The methods for this study were adapted from Bari et al. (2008) and Asinof and Paine (2014) to accommodate the 5-CSRTT for aged Long-Evans rats. Rats were acclimated to the data collection room for 1 week prior to the start of the 5-CSRTT. During acclimation, rats were food restricted until they reached 90% of free feeding weight, and feed was then adjusted with daily monitoring, so weight increased 5 g per week. The rats were subsequently acclimated to the chamber and reward pellets (Dustless Precision Pellets 45 mg, Bio-Serv, Flemington, NJ, United States) for 1 week.

After acclimation, the rats progressed through multiple training stages (see Supplementary Table S2A) based on performance for a period of 3 weeks. A rat moved to the next stage by achieving 70% correct responses on two consecutive days. Baseline testing occurred after the 3 week acclimation period and final testing occurred 5 weeks later. All rats were re-acclimated prior to the final testing period. The testing week consisted of two standard testing days, a test of attentiveness/impulsivity, and a test of cognitive function (see Supplementary Table S2B). To establish a standard performance level, each rat underwent 2 days of scored testing at the highest training stage achieved during training. To test impulsivity, the Inter-Trial Interval (ITI) was increased for each trial and all other parameters remained at those of the standard training stage. To test attentiveness, the Stimulus-Duration parameter (SD) was decreased to that of the next stage and all other parameters remained at those of the standard stage.

Nominal variables of correct, incorrect, premature, and omission were scored for each trial throughout the 4 days of testing. The two standard days of testing were averaged and used as a comparison to compute the fractional differences for the Long-ITI and Short-SD trials. Data were included for timepoints at which the rat completed an average of at least 43 trials in the standard sessions. These parameters are interpreted as measures of accuracy (fraction correct), inhibitory response control (premature), and sustained attention (omissions) (Robbins, 2002; Bari et al., 2008).

### 2.8 Statistical analysis

Linear mixed-effects models were used in a two-by-two repeated measures design with genotype (*Pink1−/−*, WT) and timepoint (baseline, final) as fixed effects and each rat as a random effect. To determine associations between behavioral and brain measures, behavior was treated as the dependent variable in a genotype X FDG uptake model. Significant interactions were followed with Tukey post-hoc analyses. All statistical analyses were performed using SAS version 9.4 (SAS Institute, Inc., Cary, North Carolina) with type III sums of squares. Significance was set at *α* = 0.05 except where otherwise noted. To qualitatively confirm genotype differences in FDG uptake found in the ROI analyses, a voxel-wise two sample *t-*test of *Pink1−/−* vs. WT images was performed and arbitrarily thresholded at |t| > 0.05 without correction for multiple comparisons.

## 3 Results

As detailed below, the main findings of this work were altered FDG uptake in several of the brain regions chosen *a priori* (PrL, Str, nucleus ambiguus, globus pallidus, caudate putamen, and posterior area of the parietal association cortex) as well as altered behavioral measures (vocalization, gait, activity, and cognition) in the *Pink1−/−* rats compared to WT controls. FDG uptake was associated with behavioral measures in several brain regions.

### 3.1 PET

#### 3.1.1 Animals scanned

Two overlapping age groups of rats (14 WT and 12 *Pink1−/−*) were scanned at two time points, with baseline and final separated by approximately 1.5 months. As detailed in Table 1, not all rats were successfully scanned at both time points, so a total of 43 scans were obtained (20 WT scans and 23 *Pink1−/−* scans).

#### 3.1.2 Image alignment and regional radioactivity time courses

**Figure 1:**
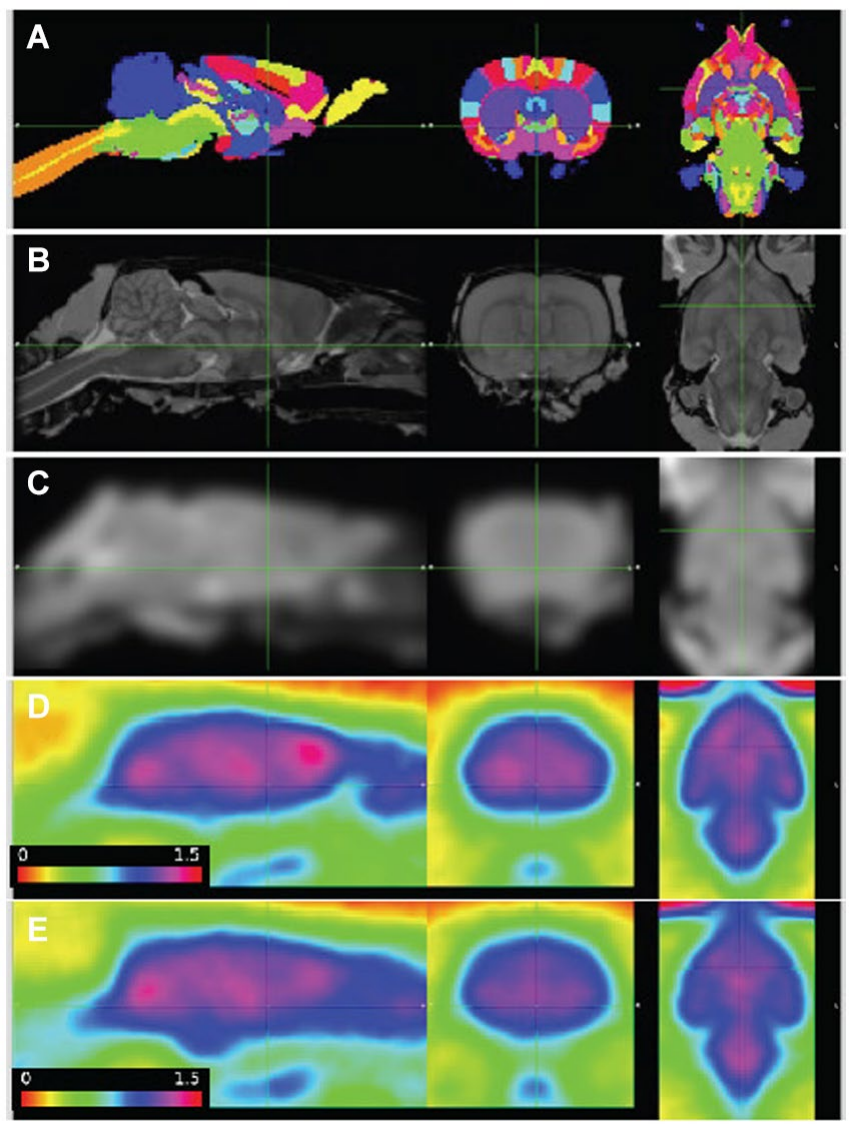
PET images aligned to atlas. **(A)** Delineated regions; **(B)** MRI template; **(C)** MRI template smoothed to PET resolution; **(D,E)** average of baseline FDG images, wildtype (*n* = 7) and *Pink1−/−* rats (*n* = 11), whole-brain normalized, 30–60 min post injection. Sagittal, coronal, and horizontal sections shown at anterior commissure.

Images were aligned to the template space with better than 1 mm accuracy (Figure 1). The combined 3-dimensional scale factors (x * y * z) of the WT image volumes at the final timepoint was 1.08 +/− 0.07 (*n* = 13, mean +/-SD), and *Pink1−/−* volumes were scaled by a lesser amount, 1.03 +/− 0.04 (*n* = 12, *p* = 0.035; two sample *t*-test). These adjustments in the brain volumes of the WT and *Pink1−/−* rats are not unexpected given that Long-Evans rats imaged here have been shown to have 4% lower brain weight compared to the Sprague Dawley rats represented in the atlas (Holson et al., 2001).

**Figure 2:**
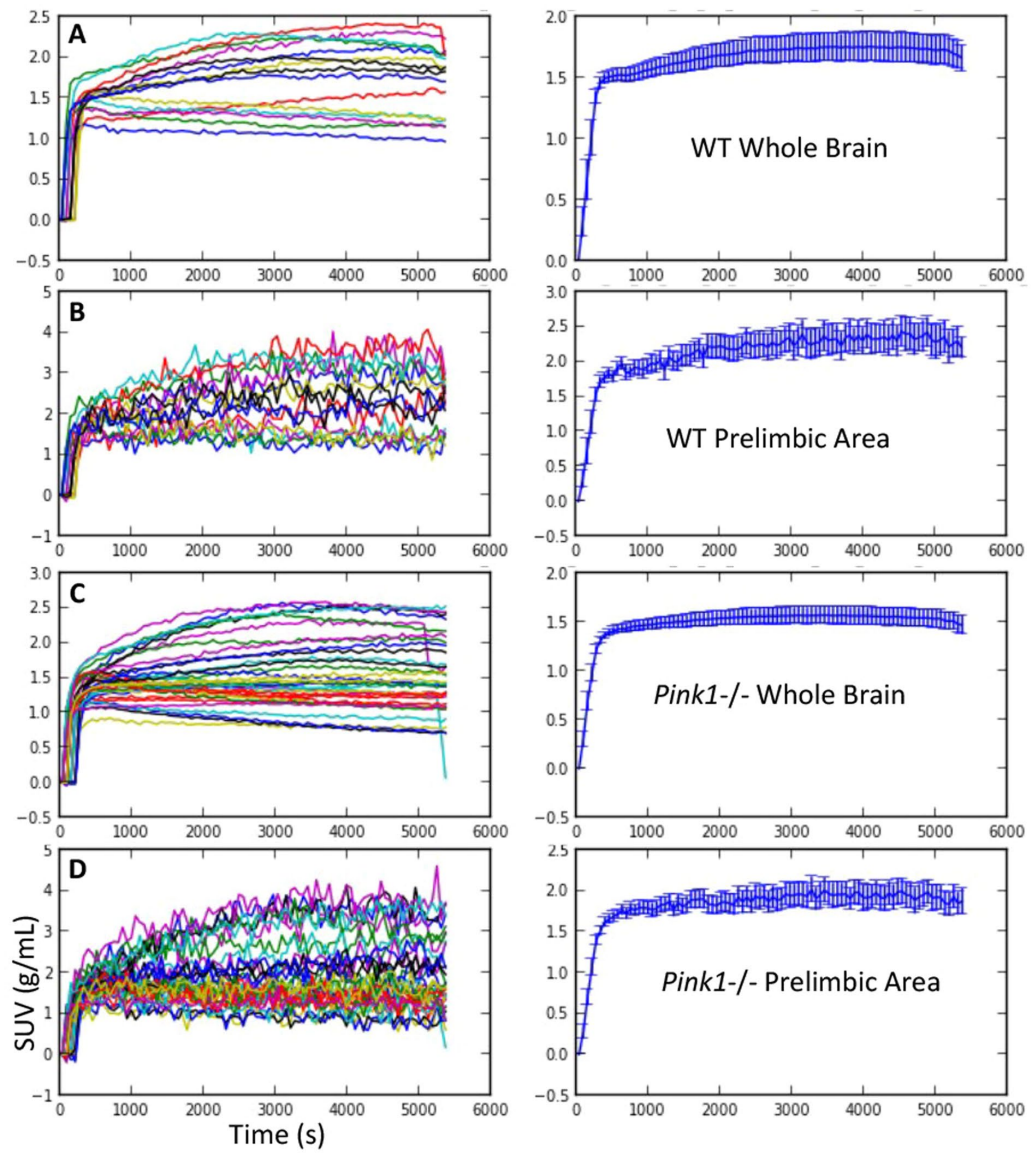
FDG uptake. Standardized uptake value (SUV) vs. time. Individual time courses are shown on the left, and mean +/− SEM are shown on the right. **(A and B)** Wildtype (WT) whole brain and prelimbic area. **(C and D)** *Pink1*−/− whole brain and prelimbic area.

Standardized uptake values (SUV) were calculated by dividing the regional radioactivity concentration 30–60 min post-injection by injected dose/body weight. For each genotype and timepoint the whole-brain SUVs were SUV_WT, baseline_ = 1.73 +/− 0.46, SUV_WT, final_ = 1.73 +/− 0.40, SUV_*Pink1−/−*, baseline_ = 1.53 +/− 0.55, and SUV_*Pink1−/−*, final_ = 1.64 +/− 0.48 (mean +/− SD). The calculated time activity curves (TACs) showed rapid uptake following IV injection of FDG and sustained radioactivity levels to the end of the 90-min scanning period. Individual and summary TACs for WT and *Pink1*−/− rats are shown in Figure 2.

#### 3.1.3 Regional whole-brain normalized FDG uptake

**Figure 3:**
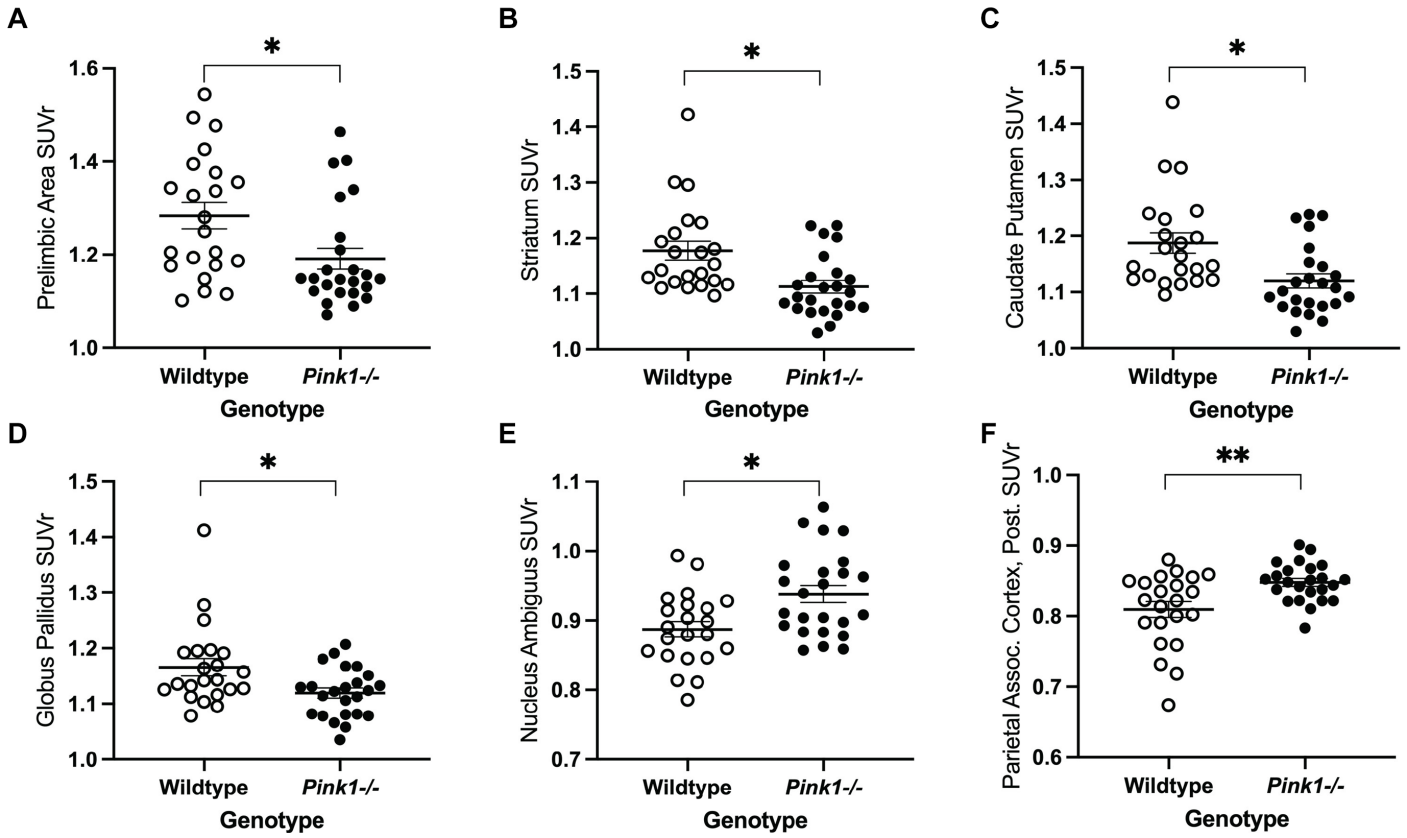
PET main effect of genotype was observed in six brain regions. Whole brain normalized FDG uptake (SUVr) was reduced in *Pink1−/−* animals in **(A)** prelimbic cortex, **(B)** striatum, **(C)** caudate putamen, and **(D)** globus pallidus, and elevated in **(E)** nucleus ambiguus and **(F)** the posterior area of the parietal association cortex. White = wildtype rats, black = *Pink1−/−* rats. Error bars indicate mean ± SEM, *p < 0.05; **p < 0.01.

**Figure 4:**
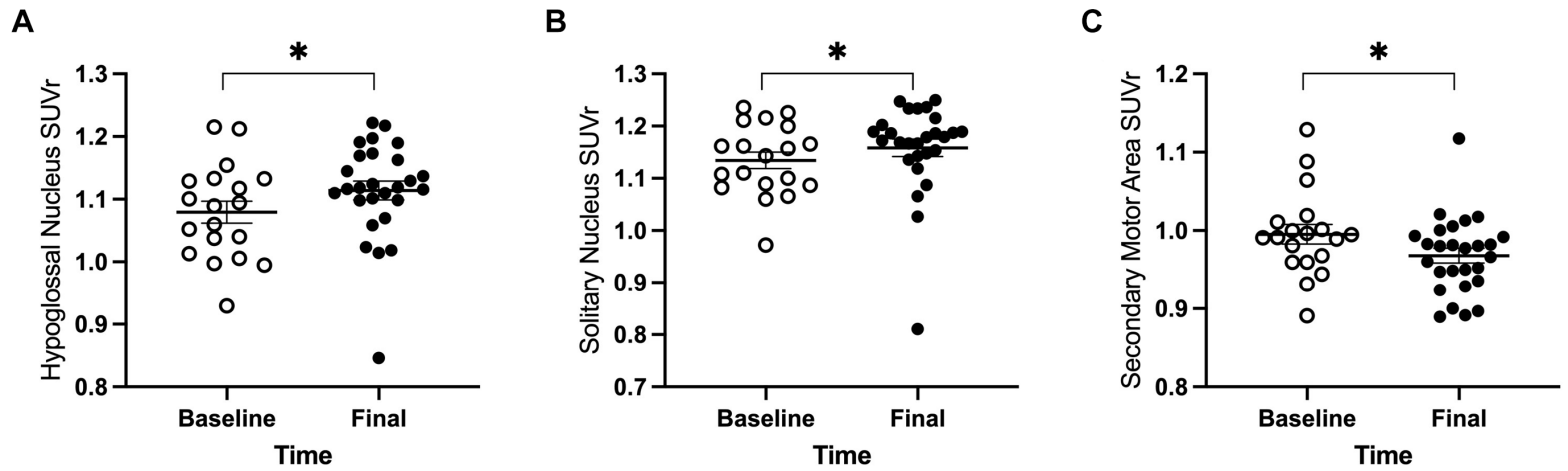
PET main effect of time was observed in three brain regions. The whole brain normalized FDG uptake (SUVr) was greater at the final timepoint in **(A)** hypoglossal nucleus and **(B)** solitary nucleus, and lower in **(C)** secondary motor area. White = baseline, black = final. Error bars indicate mean +/− SEM. *p < 0.05.

For the 18 anatomical regions of interest selected *a priori*, linear mixed-effects analyses were applied to the PET measures of relative regional FDG uptake (SUVr) with genotype and time as fixed effects and rat as a random effect. As detailed in Table 2, no interactions of genotype and time were observed. A main effect of genotype was observed in six regions (Figure 3): whole brain normalized FDG uptake was reduced in *Pink1−/−* rats in PrL [*F*(1, 24) = 6.05, *p* = 0.021], Str [*F*(1, 24) = 7.47, *p* = 0.012], caudate putamen [*F*(1, 24) = 6.87, *p* = 0.015], and globus pallidus [*F*(1,24) = 4.44, *p* = 0.046] and elevated in nucleus ambiguus [*F*(1, 24) = 6.12, *p* = 0.021] and the posterior area of the parietal association cortex [*F*(1, 24) = 8.84, *p* = 0.007]. A main effect of time was observed in three regions (Figure 4): whole brain normalized FDG uptake was greater at Final in hypoglossal nucleus (12) [*F*(1, 15) = 8.00, *p* = 0.013] and solitary nucleus [*F*(1, 15) = 4.77, *p* = 0.045] and lower in secondary motor area [*F*(1, 15) = 5.20, *p* = 0.038].

**Table 2:** PET FDG measures.

Region	Least squares means ± Standard error				Genotype ×Time			Genotype			Time		
	Baseline		Final										
	Wildtype	*Pink1−/−*	Wildtype	*Pink1−/−*	F(1,df)	df	*p*	F(1,df)	df	*p*	F(1,df)	df	*p*
Noradrenergic and dopaminergic regions													
Locus coeruleus	1.164 ± 0.031	1.173 ± 0.026	1.183 ± 0.024	1.191 ± 0.025	0.00	15	0.988	0.07	24	0.790	0.89	15	0.359
Thalamus	1.196 ± 0.015	1.170 ± 0.012	1.206 ± 0.011	1.178 ± 0.012	0.02	15	0.881	3.90	24	0.060	0.58	15	0.456
Prelimbic area	1.320 ± 0.043	1.206 ± 0.035	1.252 ± 0.032	1.179 ± 0.033	0.38	15	0.548	6.05	24	0.021*	2.00	15	0.177
Substantia nigra	0.982 ± 0.014	0.981 ± 0.011	1.001 ± 0.01	1.002 ± 0.011	0.01	15	0.929	0.00	24	0.971	2.45	15	0.138
Striatum	1.176 ± 0.022	1.116 ± 0.017	1.162 ± 0.016	1.115 ± 0.017	0.15	15	0.705	7.47	24	0.012*	0.20	15	0.662
Dysarthria regions													
Hypoglossal nucleus	1.056 ± 0.024	1.093 ± 0.019	1.110 ± 0.018	1.149 ± 0.018	0.00	15	0.967	3.43	24	0.076	8.00	15	0.013*
Solitary nucleus	1.117 ± 0.024	1.143 ± 0.019	1.168 ± 0.017	1.178 ± 0.018	0.18	15	0.679	0.83	24	0.371	4.77	15	0.045*
Nucleus ambiguus	0.890 ± 0.021	0.923 ± 0.017	0.899 ± 0.015	0.955 ± 0.016	0.45	15	0.511	6.12	24	0.021*	1.46	15	0.245
Periaqueductal gray	1.216 ± 0.022	1.199 ± 0.018	1.234 ± 0.016	1.225 ± 0.017	0.05	15	0.824	0.42	24	0.522	1.85	15	0.194
Parkinson Disease Related Pattern (PDRP) regions													
Globus pallidus	1.159 ± 0.018	1.116 ± 0.015	1.152 ± 0.013	1.125 ± 0.014	0.34	15	0.569	4.44	24	0.046*	0.01	15	0.932
Pons	1.010 ± 0.019	1.043 ± 0.016	1.029 ± 0.015	1.050 ± 0.016	0.24	15	0.631	1.74	24	0.200	1.22	15	0.287
Secondary motor area	1.016 ± 0.018	0.982 ± 0.014	0.962 ± 0.013	0.963 ± 0.014	1.22	15	0.287	1.63	24	0.213	5.20	15	0.038*
Par assoc. ctx med	0.870 ± 0.017	0.881 ± 0.014	0.879 ± 0.012	0.877 ± 0.013	0.19	15	0.673	0.11	24	0.747	0.02	15	0.894
Caudate putamen	1.187 ± 0.024	1.123 ± 0.019	1.171 ± 0.018	1.122 ± 0.018	0.18	15	0.678	6.87	24	0.015*	0.24	15	0.629
Cerebellum	0.914 ± 0.02	0.955 ± 0.016	0.952 ± 0.015	0.969 ± 0.015	0.43	15	0.522	3.26	24	0.084	2.20	15	0.159
Primary motor area	0.976 ± 0.015	0.965 ± 0.012	0.953 ± 0.011	0.947 ± 0.011	0.06	15	0.811	0.58	24	0.453	2.52	15	0.133
Par assoc. ctx post	0.812 ± 0.014	0.856 ± 0.011	0.819 ± 0.01	0.839 ± 0.011	0.84	15	0.375	8.84	24	0.007**	0.18	15	0.677
Primary visual area	0.883 ± 0.012	0.8892 ± 0.0096	0.8762 ± 0.0089	0.8728 ± 0.0093	0.20	15	0.664	0.03	24	0.858	1.06	15	0.319

Par assoc. ctx med = parietal association cortex medial area, par assoc. ctx post = parietal association cortex posterior area. Note that caudate putamen is a large sub-region of striatum. *p < 0.05, **p < 0.01.

#### 3.1.4 Brain map

**Figure 5:**
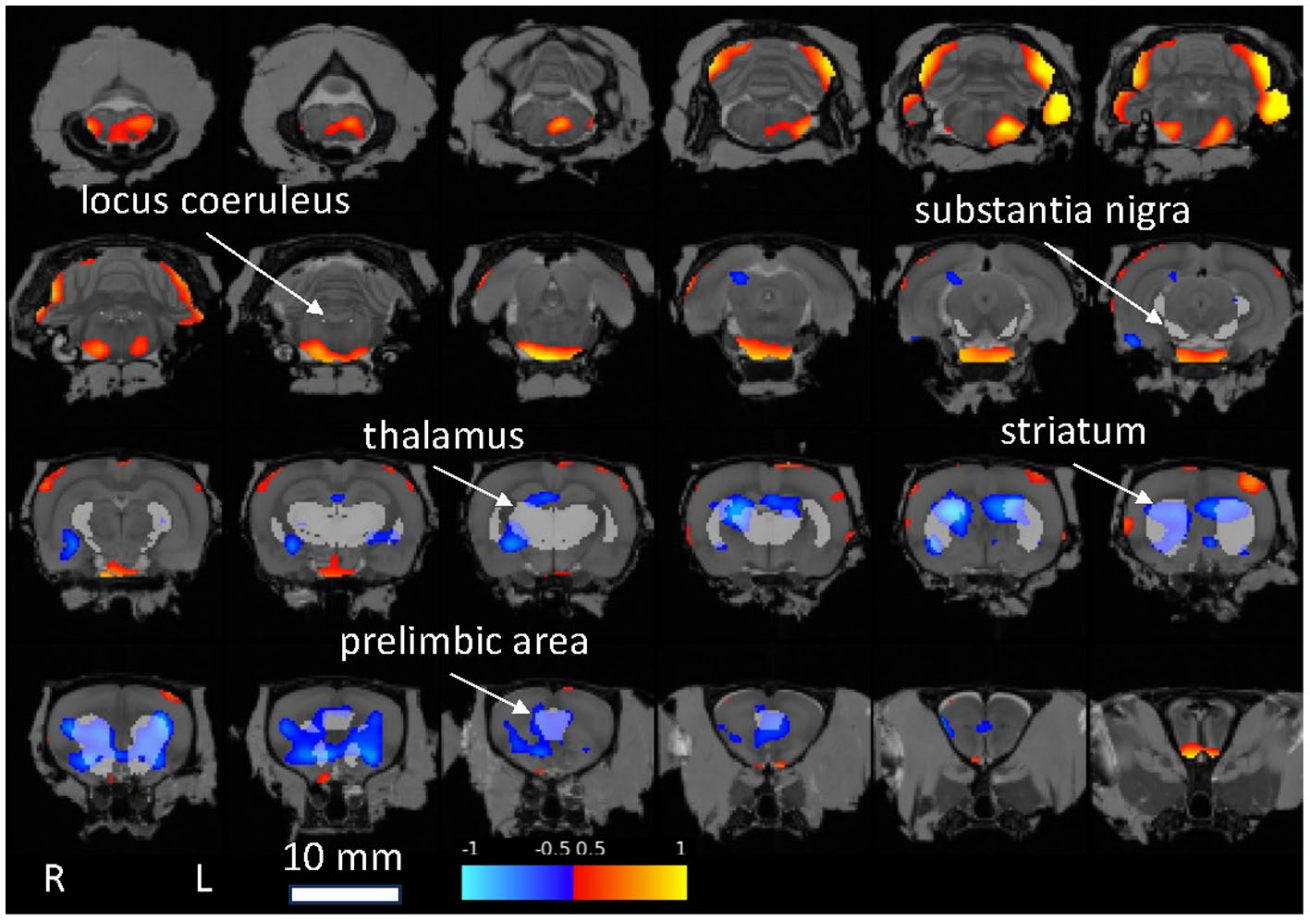
Statistical map showing *Pink1−/−* whole-brain normalized FDG uptake images compared against wildtype images (two-sample *t*-test). Red: *Pink1−/−* > wildtype; blue: *Pink1−/*− < wildtype (0.05 < |t| < 0.10); 1 mm coronal slices overlaid on MRI template image; gray regions of interest indicated by arrows are anatomically defined noradrenergic (locus coeruleus, thalamus, and prelimbic area) and dopaminergic (substantia nigra, striatum, and prelimbic area) structures.

A voxel-wise statistical map qualitatively confirmed the above ROI analyses, notably indicating reduced FDG uptake relative to whole brain in the *Pink1−/−* rats in Str and PrL (Figure 5).

### 3.2 Behavioral measures

Linear mixed-effects analyses were applied to the six ultrasonic vocalization measures, four Tapered Balance Beam measures, four Cylinder Test measures, and eight 5-CSRTT measures. As with the PET analyses, genotype and time were treated as fixed effects, and rat as a random effect. These analyses are summarized in Table 3.

**Table 3:** Behavioral measures.

Behavior	Least squares means ± Standard error				Genotype × Time			Genotype			Time		
	Baseline		Final										
	Wildtype	*Pink1−/−*	Wildtype	*Pink1−/−*	F(1,df)	df	*p*	F(1,df)	df	*p*	F(1,df)	df	*p*
Ultrasonic vocalization													
Mean power simple (dB/Hz)	−88.2 ± 1.3	−97.5 ± 1.5	−93.6 ± 1.3	−95.9 ± 1.5	8.70	24	0.007**	13.32	24	0.001**	2.69	24	0.114
Mean power FM (dB/Hz)	−87.4 ± 1.0	−96.9 ± 1.1	−93.4 ± 1.0	−95.1 ± 1.1	13.70	21	0.001**	25.30	23	0.000****	4.02	21	0.058
Peak frequency simple (kHz)	55.8 ± 1.5	55.2 ± 1.7	56.0 ± 1.5	57.4 ± 1.7	0.89	24	0.356	0.03	24	0.857	1.32	24	0.262
Peak frequency FM (kHz)	65.0 ± 1.5	61.5 ± 1.6	66.0 ± 1.5	62.3 ± 1.6	0.01	21	0.944	3.94	23	0.059	0.64	21	0.434
Bandwidth simple (kHz)	12.44 ± 0.89	11.3 ± 1.0	11.46 ± 0.89	11.7 ± 1.0	2.05	24	0.165	0.12	24	0.729	0.23	24	0.633
Bandwidth FM (kHz)	24.4 ± 1.1	15.4 ± 1.2	21.4 ± 1.1	17.5 ± 1.2	7.07	21	0.015*	23.78	23	0.000****	0.20	21	0.660
Tapered Balance Beam													
Forelimb foot faults	1.20 ± 0.24	2.22 ± 0.27	1.12 ± 0.24	1.95 ± 0.27	0.41	25	0.529	7.70	25	0.010*	1.41	25	0.247
Hindlimb foot faults	1.09 ± 0.24	2.30 ± 0.26	1.01 ± 0.24	2.43 ± 0.26	0.42	25	0.523	17.59	25	0.000***	0.03	25	0.873
Time to traverse beam (s)	3.67 ± 0.29	3.23 ± 0.32	3.64 ± 0.29	3.02 ± 0.32	0.17	25	0.680	2.09	25	0.16	0.29	25	0.598
Time to traverse final third (s)	1.87 ± 0.19	1.70 ± 0.22	1.96 ± 0.19	1.70 ± 0.22	0.08	25	0.777	0.79	25	0.382	0.08	25	0.777
Cylinder Test													
Forelimb up steps	6.5 ± 1.2	8.0 ± 1.3	4.5 ± 1.2	5.3 ± 1.3	0.09	25	0.762	0.61	25	0.442	5.84	25	0.023*
Forelimb down steps	15.7 ± 1.5	20.4 ± 1.7	14.9 ± 1.5	17.9 ± 1.7	0.24	25	0.631	6.61	25	0.016*	0.89	25	0.355
Hindlimb steps	15.9 ± 1.5	22.8 ± 1.7	10.7 ± 1.5	18.9 ± 1.7	0.22	25	0.644	17.97	25	0.000***	9.58	25	0.005**
Rears	10.6 ± 0.9	15.2 ± 1.0	8.87 ± 0.90	13.2 ± 1.0	0.01	25	0.918	19.33	25	0.000***	4.27	25	0.049*
Lands	9.93 ± 0.88	15.00 ± 0.98	8.53 ± 0.88	13.00 ± 0.98	0.11	25	0.741	24.29	25	0.000****	3.58	25	0.070
Five-Choice Serial Reaction Time Task (5CSRTT)													
Long ITI ΔCorrect (%)	−11.8 ± 6.7	1.7 ± 4.7	−16.9 ± 5.5	−4.7 ± 4.7	0.03	5	0.865	3.46	9	0.096	2.80	5	0.155
Long ITI ΔIncorrect (%)	−0.3 ± 3.1	−0.2 ± 1.8	−2.4 ± 2.2	3.5 ± 1.8	1.69	5	0.251	1.67	9	0.228	0.11	5	0.750
Long ITI ΔPremature (%)	18 ± 12	29.1 ± 7.1	7.7 ± 8.6	25.9 ± 7.1	0.22	5	0.661	2.17	9	0.175	0.78	5	0.417
Long ITI ΔOmission (%)	11.7 ± 5.7	−1.5 ± 4.3	19.5 ± 4.9	1.2 ± 4.3	0.97	5	0.370	6.26	9	0.034*	4.18	5	0.096
Short SD ΔCorrect (%)	−3.2 ± 9.3	−1.7 ± 5.5	−32.4 ± 6.7	−29.1 ± 5.5	0.02	5	0.885	0.10	9	0.762	21.78	5	0.005**
Short SD ΔIncorrect (%)	−0.9 ± 4.4	0.7 ± 2.6	1.1 ± 3.1	7.2 ± 2.6	0.46	5	0.530	1.46	9	0.258	1.60	5	0.261
Short SD ΔPremature (%)	4.8 ± 9.4	−3.6 ± 5.6	−2.2 ± 6.7	6.1 ± 5.6	1.74	5	0.244	0.00	9	0.993	0.05	5	0.834
Short SD ΔOmission (%)	20.0 ± 9.2	1.1 ± 5.7	32.6 ± 6.8	21.9 ± 5.7	0.74	5	0.429	0.51	9	0.494	20.73	5	0.006**

*p < 0.05, **p < 0.01, ***p < 0.001, ****p < 0.0001.

#### 3.2.1 Ultrasonic vocalizations

**Figure 6:**
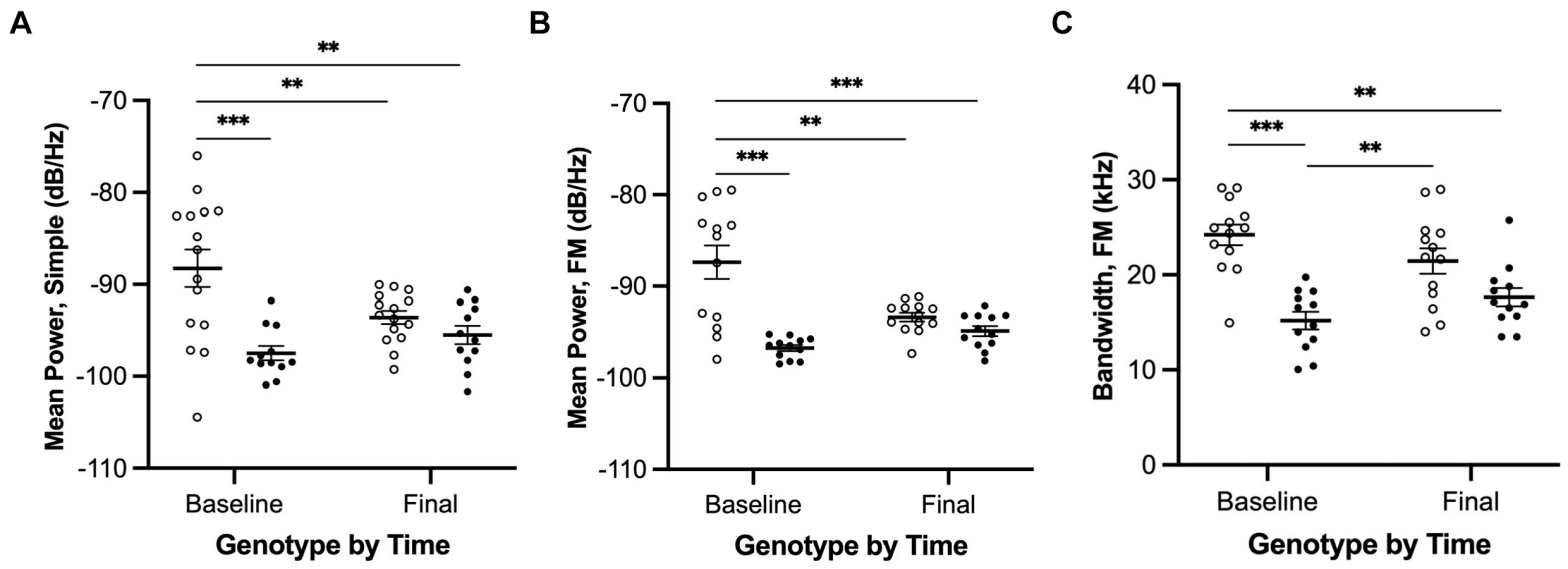
Ultrasonic vocalizations interactions of genotype and time for simple and FM calls. **(A)** Mean power of simple calls, **(B)** Mean power of FM calls, and **(C)** Bandwidth of FM calls. White = wildtype rats; black = *Pink1−/−* rats. Error bars indicate mean +/− SEM. **p < 0.01, ***p < 0.001.

Interactions of genotype and time were observed in three of the USV measures (mean power of simple calls, mean power of FM calls, and bandwidth of FM calls) as illustrated in Figure 6 and detailed here.

We observed a significant interaction effect between genotype and time for mean power of simple calls [*F*(1, 24) = 8.7, *p* = 0.007, Figure 6A]. Pairwise comparisons revealed that (1) WTs at baseline produced simple calls with greater mean power than WTs at final (*p* = 0.0087), (2) WTs at baseline produced simple calls with greater mean power than *Pink1−/−* rats at baseline (*p* = 0.0005), and (3) WTs at baseline produced simple calls with greater mean power than *Pink1−/−* rats at final (*p* = 0.0034).

We observed a significant interaction effect between genotype and time for mean power of FM calls [*F*(1, 21) = 13.7, *p* = 0.0013, Figure 6B]. Pairwise comparisons revealed that (1) WTs at baseline produced FM calls with greater mean power than WTs at final (*p* = 0.0021), (2) WTs at baseline produced FM calls with greater mean power than *Pink1−/−* rats at baseline (*p* < 0.0001), and (3) WTs at baseline produced FM calls with greater mean power than *Pink1−/−* rats at final (*p* = 0.0003).

We observed a significant interaction effect between time and genotype for bandwidth of FM calls [*F*(1, 21) = 7.07, *p* = 0.0147, Figure 6C]. Pairwise comparisons revealed that (1) WTs at baseline produced FM calls with greater bandwidth than *Pink1−/−* rats at baseline (*p* = 0.0001), (2) WTs at baseline produced FM calls with greater bandwidth than *Pink1−/−* rats at final (*p* = 0.0021), and (3) *Pink1−/−* rats at baseline produced FM calls with lower bandwidth than WTs at final (*p* = 0.0069).

#### 3.2.2 Tapered Balance Beam

**Figure 7:**
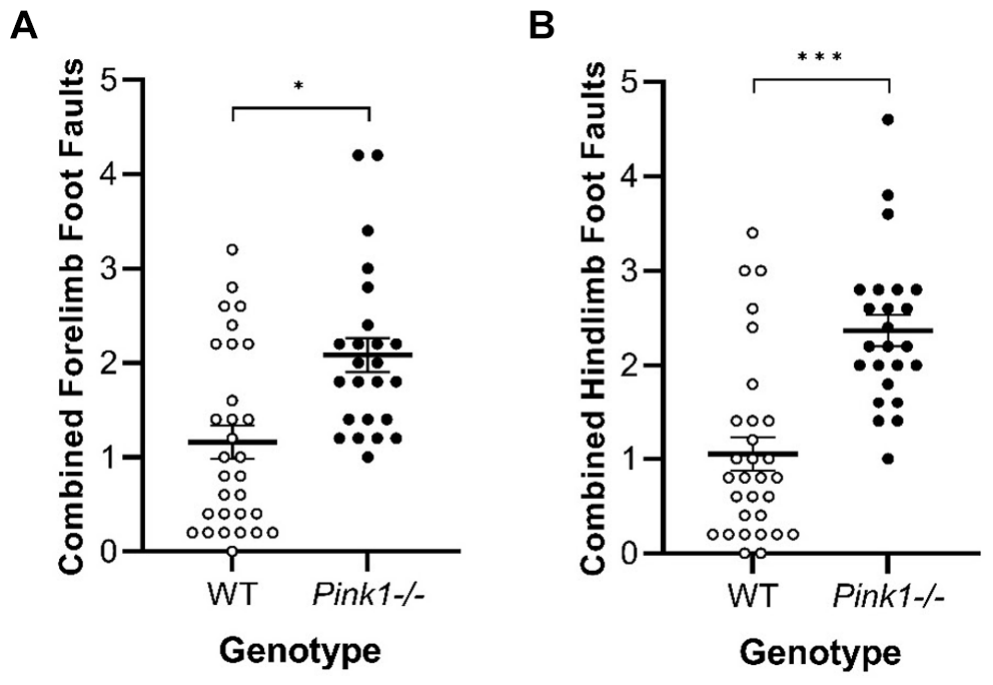
Tapered Balance Beam main effect of genotype. *Pink1−/−* rats exhibited more **(A)** forelimb foot faults and **(B)** hindlimb foot faults. White = wildtype rats; black = *Pink1−/−* rats. Error bars indicate mean +/− SEM. *p < 0.05; ***p < 0.001.

No interactions of genotype and time were observed. A main effect of genotype was observed in two of the Tapered Balance Beam measures (Figure 7): *Pink1−/−* rats exhibited greater forelimb [*F*(1, 25) = 7.70, *p* = 0.01] and greater hindlimb [*F*(1, 25) = 17.59, *p* < 0.001] foot faults.

#### 3.2.3 Cylinder Test

**Figure 8:**
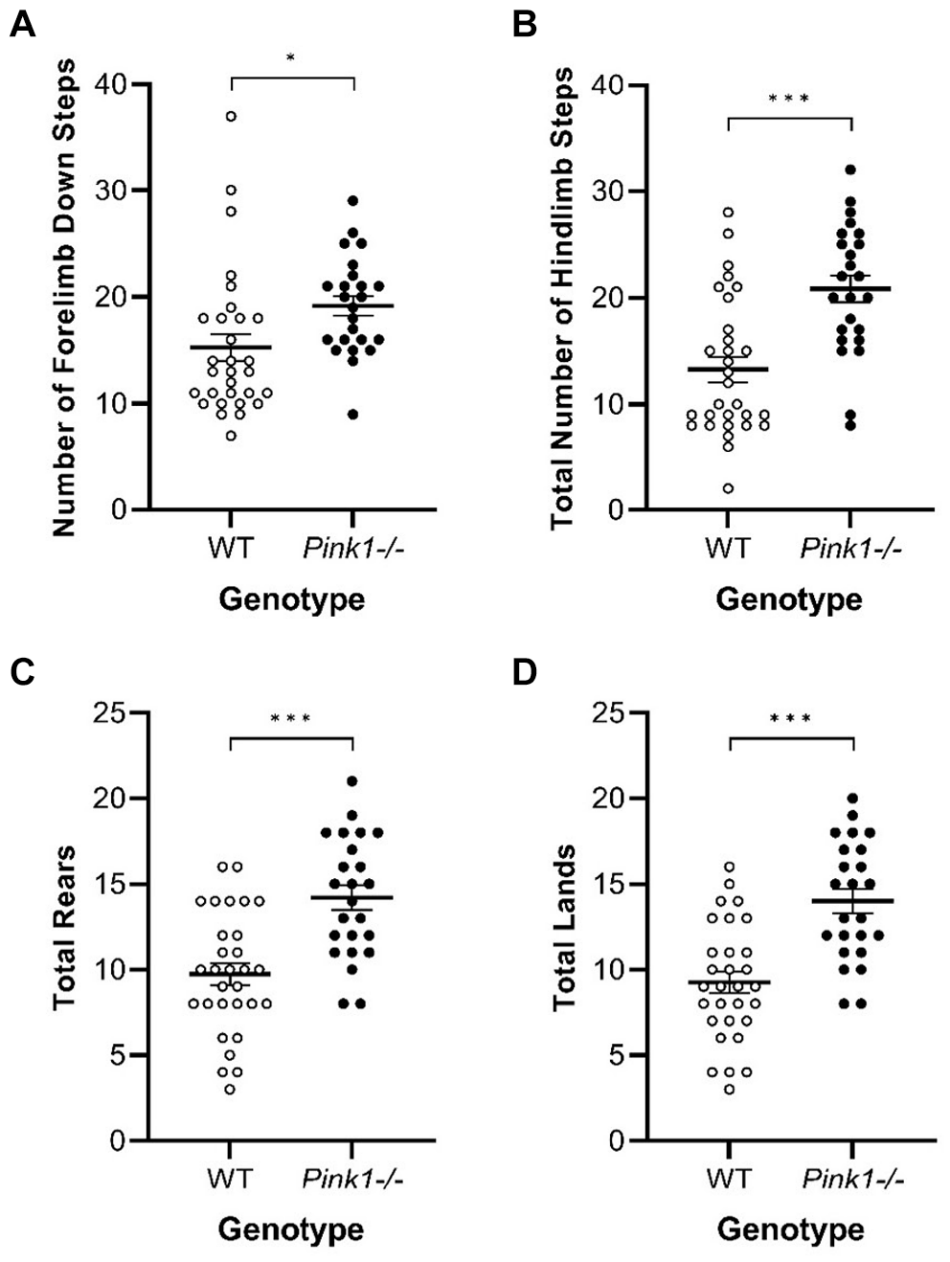
Cylinder Test main effects of genotype. *Pink1−/−* animals exhibited greater numbers of **(A)** forelimb down steps, **(B)** hindlimb steps, **(C)** rears, and **(D)** lands. White = wildtype controls, black = *Pink1−/−* rats. Error bars indicate mean +/− SEM. *p < 0.05; ***p < 0.001.

**Figure 9:**
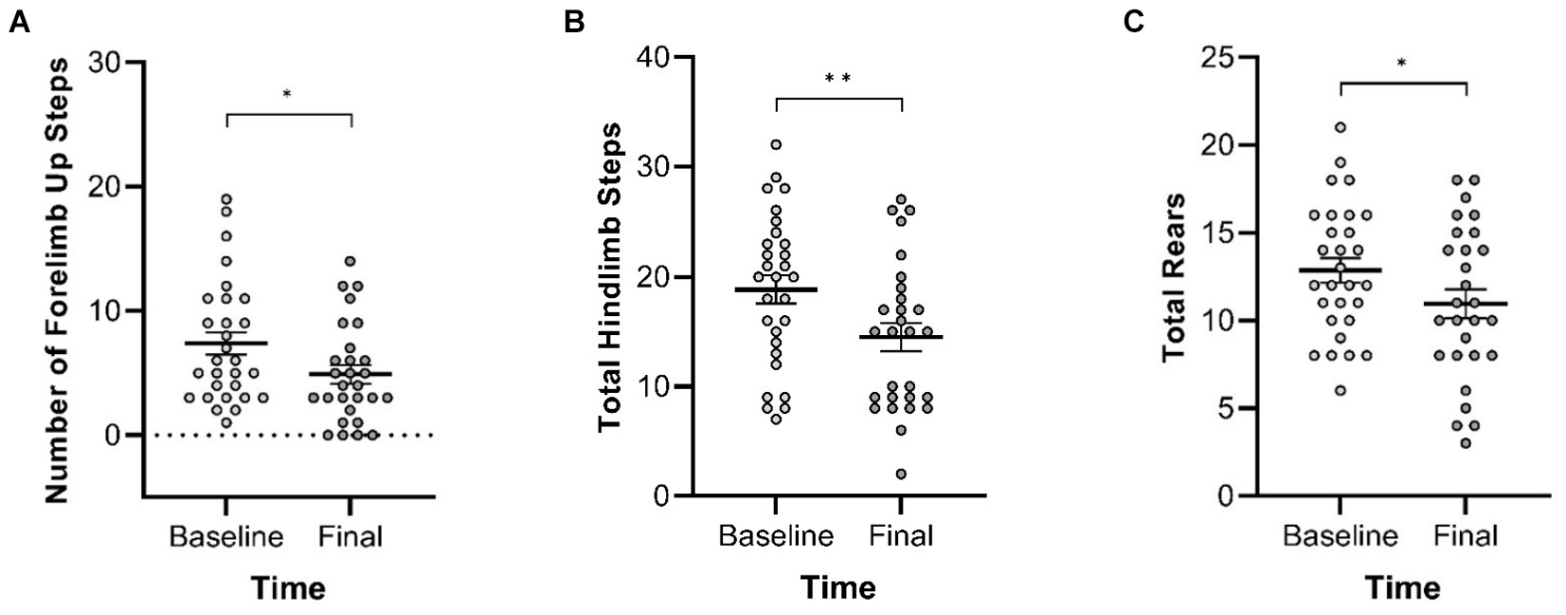
Cylinder Test main effects of time. At the final timepoint, rats exhibited reduced numbers of **(A)** forelimb up steps, **(B)** hindlimb steps, and **(C)** rears. White = baseline; gray = final. Error bars indicate mean +/− SEM. *p < 0.05; **p < 0.01.

In the Cylinder Test, no interactions of genotype and time were observed. A main effect of genotype was observed in four of the measures (Figure 8) with *Pink1−/−* rats exhibiting greater numbers of forelimb down steps [*F*(1, 25) = 6.61, *p* = 0.016], hindlimb steps [*F*(1, 25) = 17.97, *p* < 0.001], rears [*F*(1, 25) = 19.33, *p* < 0.001], and lands. A main effect of time was observed with rats exhibiting fewer forelimb up steps, hindlimb steps, and rears at Final (Figure 9).

#### 3.2.4 Five-Choice Serial Reaction Time Task

**Figure 10:**
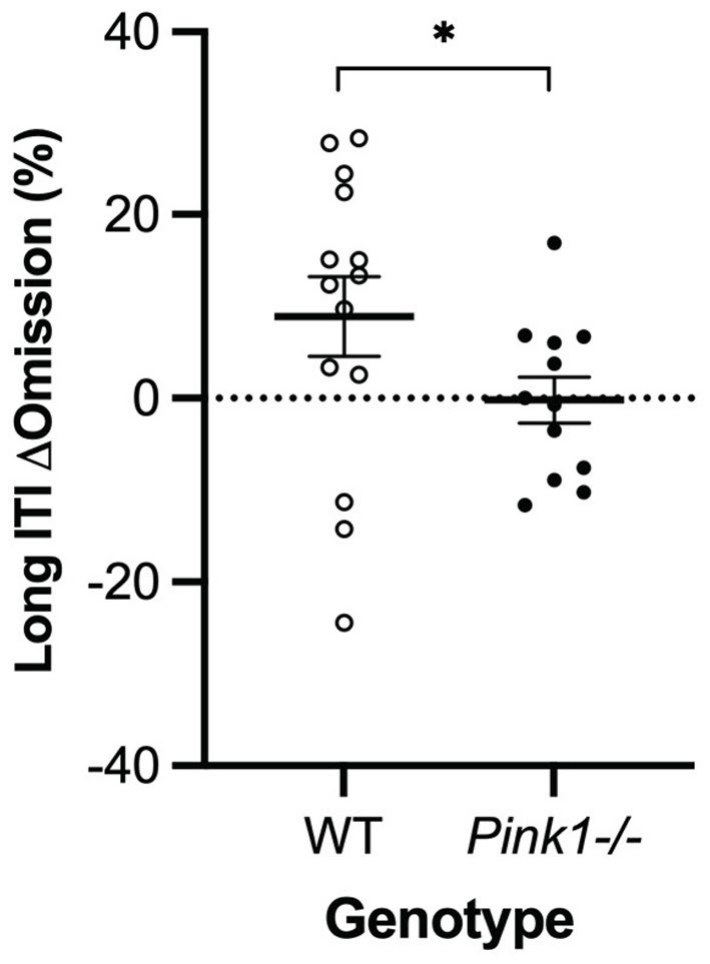
Five-Choice Serial Reaction Time Task long inter-trial interval main effect of genotype. *Pink1−/−* rats exhibited fewer omissions relative to standard trials. White = wildtype rats; black = *Pink1−/−* rats. Error bars indicate mean +/− SEM. *p < 0.05.

**Figure 11:**
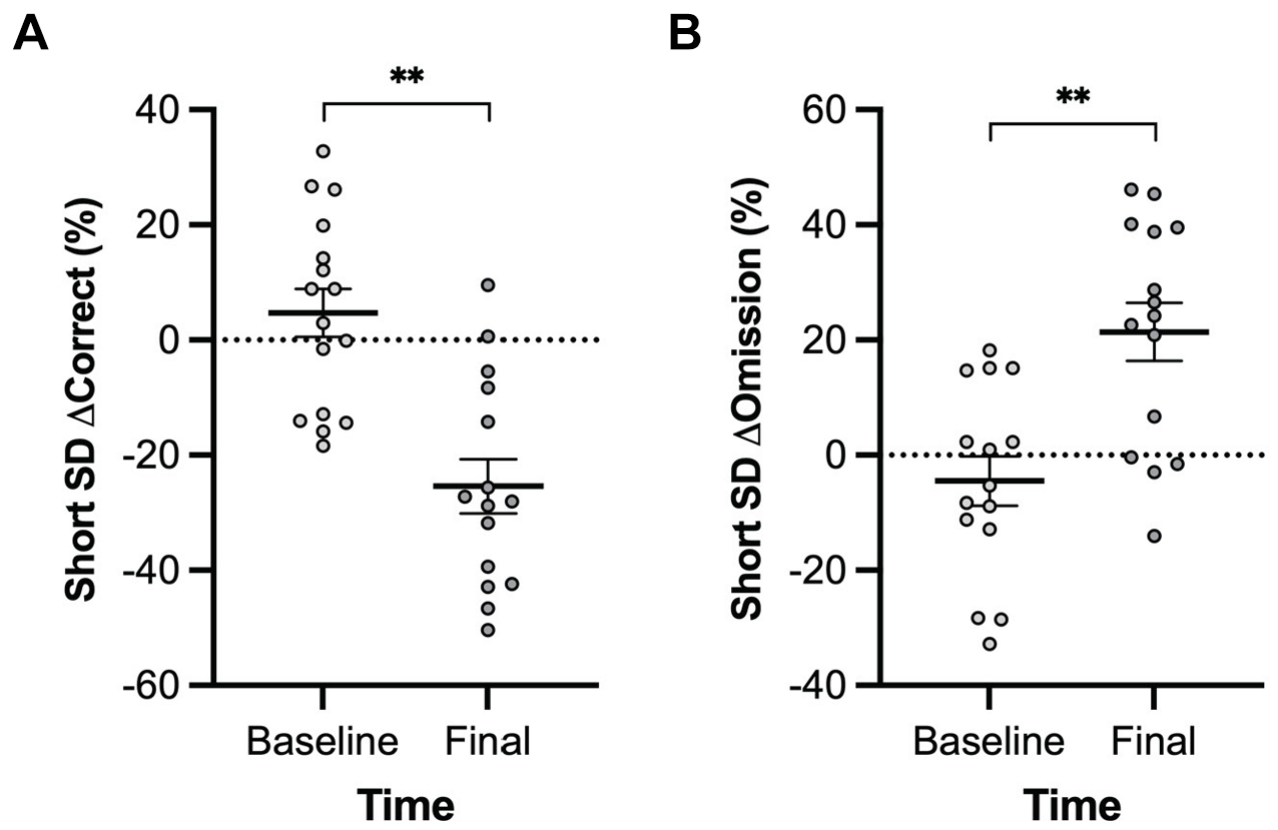
Five-Choice Serial Reaction Time Task short stimulus duration main effect of time. Rats at the final timepoint exhibited **(A)** fewer correct responses and **(B)** more omissions relative to standard trials. White = baseline; black = final. Error bars indicate mean +/− SEM. **p < 0.01.

A main effect of genotype was observed in the long inter-trial interval omission delta score with *Pink1−/−* rats exhibiting fewer additional omissions relative to standard trials [*F*(1, 9) = 6.26, *p* = 0.034] (Figure 10). Also, main effects of time were observed in short stimulus display delta scores with rats at Final exhibiting fewer correct responses [*F*(1, 5) = 21.78, *p* = 0.005] and more omissions [*F*(1, 5) = 20.73, *p* = 0.006] (Figure 11).

### 3.3 Relationships between behavioral and PET measures

**Figure 12:**
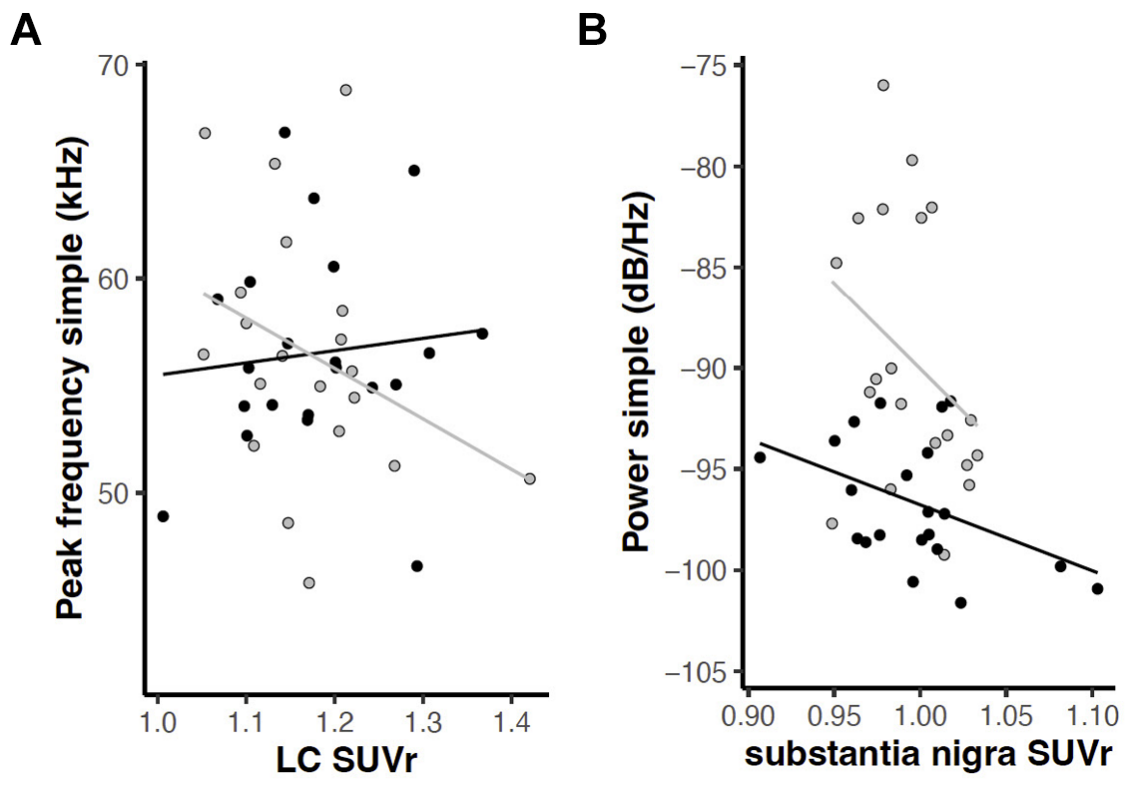
Relations between USV parameters and FDG uptake in catecholaminergic regions (*p* < 0.05). **(A)** Interaction of region and genotype in relation of peak frequency of simple calls and locus coeruleus. **(B)** Main effect of brain region in relation of mean power of simple calls to substantia nigra. Gray = wildtype rats; black = *Pink1−/−* rats. Lines indicate linear regression by genotype.

To examine correlations between behavioral and PET measures, linear mixed effects analyses were applied with behavior as the dependent variable described by a regional FDG uptake x genotype model. Given our overarching interest in norepinephrine, dopamine, and dysarthria, we focused *a priori* within this exploration on relationships between the six USV measures (mean power, peak frequency, and bandwidth of simple and FM calls) and FDG uptake in the five catecholaminergic regions (LC, Thal, PrL, SN, and Str). An interaction effect (region and genotype) relating peak frequency in simple calls to LC [*F*(1, 14) = 5.14, *p* = 0.040] and a main effect of brain region relating mean power of simple calls to SN [*F*(1, 14) = 5.22, *p* = 0.038] were revealed (Figure 12).

Exploring relations between all 23 behavioral measures and 18 brain regions involved 414 tests. Of these, 29 tests yielded significant interactions of region and genotype, and 20 tests yielded significant main effects of region at *p* < 0.05 (Supplementary Tables S3, S4). To further focus this exploratory analysis, significance was limited to *p* < 0.005, which two tests would be expected to meet by chance. This yielded five significant interactions of region and genotype, in which *Pink1−/−* rats exhibited different dependencies of behavior on FDG uptake compared to WT (Supplementary Figure S1). Interactions were observed relating 5-CSRTT long ITI premature responses to nucleus ambiguus [*F*(1, 10) = 13.83, *p* = 0.004], Cylinder Test rears to medial parietal cortex [*F*(1, 15) = 13.73, *p* = 0.002], Cylinder Test lands to medial parietal cortex [*F*(1, 15) = 15.37, *p* = 0.001], USV FM calls peak frequency to cerebellum [*F*(1, 14) = 11.52, *p* = 0.004], and USV FM calls peak frequency to motor cortex [*F*(1, 14) = 18.75, *p* = 0.0007]. Additionally at p < 0.005 there were two significant main effects of region (Supplementary Figure S2) relating 5-CSRTT long ITI incorrect responses to nucleus ambiguus [*F*(1, 10) = 14.14, *p* = 0.004] and Cylinder Test forelimb down to medial parietal cortex [*F*(1, 15) = 12.24, *p* = 0.003].

## 4 Discussion

### 4.1 FDG PET

This novel application of FDG PET imaging to the *Pink1−/−* rat model of PD revealed abnormalities in relative glucose metabolism in several regions of interest identified *a priori* (Table 2) for their associations with catecholaminergic circuits, vocalization, cognition, limb use, and PD (Hypothesis 1).

We observed reduced FDG uptake in *Pink1−/−* rats in two of the five regions that we focused on because of their roles in the dopaminergic and noradrenergic systems, namely PrL and Str. PrL and Str are rich in dopamine receptors and are involved in motor function regulation (Bamford and Bamford, 2019). As dopaminergic neurons degenerate in PD, there is a change in metabolic activity and glucose utilization in these areas, reflected by altered FDG uptake in PET imaging studies (Eidelberg, 2009; Eggers et al., 2014; Liu et al., 2018; Seiffert et al., 2022). Additionally, in rats PrL is innervated by noradrenergic axons, and degeneration of such projections to homologous regions in PD may contribute to cognitive decline (Miner et al., 2003).

In this study we also found elevated FDG uptake in *Pink1−/−* rats in the nucleus ambiguus and the posterior area of the parietal association cortex. Elevated FDG uptake in certain brain regions can indicate increased metabolic activity and glucose utilization in those areas. This heightened uptake often reflects physiological or pathological processes such as neuronal activation, synaptic transmission, neuroplasticity, or compensatory mechanisms. In disease, compensatory mechanisms or neuroinflammatory responses may lead to increased glucose metabolism and subsequent FDG uptake in specific brain regions (Teune et al., 2010). The nucleus ambiguus, involved in voice, swallowing, and autonomic functions, may exhibit increased metabolic activity as a compensatory response to dysfunction in other brain regions affected by PD pathology (Schapira et al., 2017; Coon et al., 2018). Moreover, the nucleus ambiguus is innervated by the vagus nerve, which may be one of the earliest implicated pathways in the pathogenesis of PD (Braak et al., 2003; Hawkes et al., 2010; Goedert et al., 2013). The posterior area of the parietal association cortex also showed elevated FDG uptake in this study; this area plays a role in sensory integration and spatial awareness, and may also be compensating for deficits in motor control and sensory processing associated with PD (Isaias et al., 2020). This is in line with the *decreased* FDG uptake we observed in *Pink1−/−* rat brain regions involved in motor functioning (i.e., PrL and Str). Enhanced FDG uptake in these regions may reflect adaptive responses aimed at maintaining functional integrity despite dopaminergic neuronal loss or other changes in the pathogenesis of PD.

Contrary to our expectations, we did not observe metabolic differences in LC, despite evidence of altered norepinephrine, tyrosine hydroxylase, and α_1_ receptors in this structure as reported in our previous work (Kelm-Nelson et al., 2018; Hoffmeister et al., 2021). FDG-PET imaging primarily reflects glucose metabolism and overall neural activity, which may not always directly correlate with specific neurotransmitter changes or receptor alterations. Additionally, changes in norepinephrine levels or receptor expression in the LC may occur without significant alterations in glucose metabolism or FDG uptake, particularly when compensatory mechanisms may be at play. The relationship between catecholamines and metabolic activity in the LC is complex and may involve multiple regulatory mechanisms beyond just glucose metabolism. Therefore, while changes in norepinephrine, tyrosine hydroxylase immunoreactivity, and α_1_ receptors have been observed, these alterations may not always manifest as detectable changes in FDG uptake in PET imaging studies. Further studies using more specific radiotracers may be helpful to illuminate the roles of dopamine and norepinephrine in *Pink1−/−* rats.

Regarding the effects of timepoint (Hypothesis 4) in the FDG PET results, we observed no significant difference in the rate of change between *Pink1−/−* and WT rats, i.e., no interactions of genotype and timepoint. We did, however, observe main effects of timepoint with increased uptake in hypoglossal nucleus and solitary nucleus at final compared to baseline timepoints and decreased uptake in the secondary motor area. These results may reflect changes with age common to both *Pink1−/−* and WT rats. Six weeks between time points within subject may not have been sufficient to observe additional neurological changes.

### 4.2 Behavior

We hypothesized a decline in behavioral function in *Pink1−/−* rats compared to WT rats (Hypothesis 2), and our analyses revealed significant differences in ultrasonic vocalizations, motor performance and coordination, spontaneous motor activity, and cognitive behavior.

#### 4.2.1 Ultrasonic vocalizations

Vocal deficits, dysarthria, and motor speech disorders are common signs during the course of PD and greatly impact vocal communication (Tjaden, 2008; Liss et al., 2009; Bowen et al., 2013; Ciucci et al., 2013; Stepp, 2013; Di Pietro et al., 2022; Rohl et al., 2022). Common vocal deficits are decreased loudness, monotone pitch, and decreased intelligibility (Sapir et al., 2008; Plowman-Prine et al., 2009). The *Pink1−/−* rat model of PD has been used in the past to investigate vocal deficits (Krasko et al., 2021). Studies revealed that vocal intensity was the most affected acoustic outcome, followed by reduced bandwidth of vocalizations (up to 10 months of age). These findings are highly analogous to clinically presenting reduced loudness and range in patients with PD (for review see Krasko et al., 2021).

In our study, mean power of simple calls and FM calls was reduced at baseline between WT and *Pink1−/−* rats (Simple∆: −9.22 dB/Hz; FM∆: −9.48 dB/Hz). We furthermore found reduced bandwidth in FM calls between *Pink1−/−* and WT at baseline (∆: −8.96 kHz), confirming findings from previous studies that investigated early stages of PD (Grant et al., 2015; Krasko et al., 2021). Interestingly, we also found a reduction in mean power in WT rats from baseline to the final timepoint for both simple (∆: 6.05 dB/Hz) and FM (∆: 5.33 dB/Hz) vocalizations, but we could not confirm this for *Pink1−/−* rats: While we do see a distinct difference in mean power and bandwidth between genotypes at baseline, only WT vocalizations show a decline in mean power over time while *Pink1−/−* rats remain in about the same intensity range. This significant decrease in mean power in WT rat vocalizations could be attributed to aging, which also has a significant effect on call intensity (Basken et al., 2012), or it could be an effect of habituation and loss of motivation/interest. Here, *Pink 1−/−* rats might not display pronounced aging effects due to their already heavily impacted mean power at baseline (Simple: −97.45 dB/Hz; FM: −96.86 dB/Hz). It is important to note that our rats varied in age at each timepoint, and testing occurred twice (baseline/final) with all rats generally ranging from 9 to 11 months of age across timepoints, which could have impacted our findings. In a study by Johnson and colleagues, *increased* intensity in *Pink1−/−* rats has been described at around 10 months of age with mean power otherwise declining up to this specific timepoint, which has been found in conjunction with increased motor variability at around 10 months of age in *Pink1−/−* rats (Johnson et al., 2020). The timepoints we chose for this study might have captured a transitioning period that ultimately resulted in no significant differences between baseline and final timepoints in *Pink1−/−* rats. The decreasing aging/habituation effect on mean power and the increasing disease-related effect on mean power (which typically occurs for *Pink1−/−* rats at 10-months of age) might have resulted in an overall counter-balancing effect, explaining the steady range of mean power between timepoints in *Pink 1−/−* rats in our results.

#### 4.2.2 Tapered Balance Beam

Two major hallmark signs in PD are the effects of the disease on motor performance and coordination with more pronounced deficits in motor function and coordination as the disease progresses (Mazzoni et al., 2012; Antony et al., 2013; Moustafa et al., 2016). In this study, we found that motor performance and coordination was impaired in *Pink1−/−* rats: *Pink1−/−* rats exhibited a greater number of forelimb and hindlimb foot faults compared to WT controls. Our findings confirm the presence of gross motor deficits that have been described in previous studies using this rat model (Dave et al., 2014; Grant et al., 2015; Marquis et al., 2020; Kelm-Nelson et al., 2021) and similar timepoints (Johnson et al., 2020). The observed motor performance and coordination deficits in the Tapered Balance Beam task did not differ across time, which could be a result of the chosen timepoints where motor deficits might already be pronounced, and further deficiencies might only appear subtly, or the short time period in between tests may not have been sufficient to note significant motor decline.

#### 4.2.3 Cylinder Test

Spontaneous activity was tested via Cylinder Test, and *Pink1−/−* rats performed more forelimb down steps, hindlimb steps, and rears compared to WT rats. This is consistent with previous findings where 8-month-old *Pink1−/−* rats demonstrated higher spontaneous activity with an increase in hindlimb movements than WT rats (Grant et al., 2015); another study reported more forelimb movements in young adult *Pink1−/−* rats when compared with WT rats, without any significant sex differences (Lechner et al., 2022). We also found an overall decrease in the number of forelimb steps, hindlimb steps, and rears over time for both *Pink1−/−* and WT rats indicating that, regardless of genotype, rats move less as they age. This has previously been observed in female *Pink1−/−* and female WT rats (Marquis et al., 2020).

#### 4.2.4 Five-Choice Serial Reaction Time Task

In this novel application of the Five-Choice Serial Reaction Time Task with the *Pink1−/−* rat model, we found a smaller number of omissions in the long inter trial interval (ITI) trials relative to standard trials when compared with WT omissions for the same task. *Prima facie*, this might be interpreted as better attention in the *Pink1−/−* rats, but this seems unlikely, and it may instead be a consequence of adaptation to the task in the WT controls (Asinof and Paine, 2014). We also observed a main effect of time in short stimulus display delta scores with rats at the final timepoint exhibiting fewer correct responses and more omissions relative to standard trials regardless of genotype when compared to the baseline scores. This could be an indicator of reduced attention with time, perhaps due to effects of aging or adaptation common to both genotypes (Asinof and Paine, 2014). Due to logistical issues, a subset of only 6 *Pink1−/−* and 4 WT rats were successfully assessed with the 5-CSRTT, and further work with this test in this model may be warranted.

### 4.3 Brain-behavior associations

In this study we observed associations between behavioral and PET measures in several brain regions (Hypothesis 3). Notably, relationships were found between vocalization parameters and FDG uptake in LC and SN, regions rich in noradrenergic and dopaminergic cell bodies, respectively. In *Pink1−/−* rats, the peak frequency of simple calls was seen to increase with LC metabolism, while WT calls decreased with LC metabolism. This may reflect inflammation or compensatory hypermetabolism in LC, where we previously found reduced norepinephrine levels in *Pink1−/−* rats (Kelm-Nelson et al., 2018). Substantia nigra and nigrostriatal pathway integrity are necessary for 50 kHz vocalization in rats, as has been demonstrated by 6-OHDA infusion in SN and, unilaterally, the medial forebrain bundle (Vecchia et al., 2018; Simola et al., 2021). In this study, the mean power of simple calls decreased with FDG uptake in SN in both *Pink1−/−* and WT rats. The latter finding suggests that SN integrity or function may be involved in the reduced simple call power we observed in this study in *Pink1−/−* rats, perhaps reflective of inflammation or a compensatory response to losses elsewhere.

This study revealed genotype differences in several other relationships between behavioral measures and FDG uptake. For example, *Pink1−/−* rats had positive relationships between rears/lands and FDG uptake in the medial parietal association cortex, while WT rats showed negative relationships for these brain-behaviors. As a compensatory response, *Pink1−/−* rats may exhibit increased FDG uptake in certain brain regions, including the medial parietal association cortex, as the brain attempts to recruit additional resources or alternative pathways to compensate for impaired function (Reuter-Lorenz and Cappell, 2008; Fettrow et al., 2021). The positive relationship observed between rears/lands and FDG uptake in the medial parietal association cortex in *Pink1−/−* rats may reflect this compensatory mechanism. In PD, higher FDG uptake in the medial parietal association cortex could indicate increased neural activity or recruitment of additional motor planning resources in response to gait disturbances. This suggests that PD patients may rely more heavily on cortical areas, including the medial parietal association cortex, to compensate for motor deficits. It should be noted, however, that while the increased rears/lands in *Pink1−/−* rats is interpreted as gait instability, it cannot be stated with certainty that decreased rears/lands in WT rats is an indication of gait stability. This is because we did not make a distinction between time maintaining a rear, and time remaining on all fours; both scenarios would result in decreased rears/lands.

### 4.4 Limitations

This study was subject to certain limitations. Partial volume effects due to the finite spatial resolution of the PET scanner (2 mm full width at half maximum) limited the certainty of anatomical identification. Due to scanner downtime, the younger group of WT rats were scanned only at the final timepoint. This may have reduced sensitivity to neurodegeneration (genotype × timepoint), especially in the ~9.2 to ~10.6 months sub-range. Further, the relatively short period of time in between testing periods (6 weeks) may not have been sufficient to capture robust neurodegeneration within each sub-range, and variance between the rats in the two sub-ranges may have obscured changes over the combined 9–12 month age range. Typically, studies from our lab test at least 8–12 weeks in between time points. Finally, comparisons of these experimental animal findings and clinical observations of FDG uptake may be complicated by the effects on glucose metabolism of drugs such as L-DOPA in patients and isoflurane anesthesia in rats (Matsumura et al., 2003; Ko et al., 2015; Spangler-Bickell et al., 2016).

## 5 Conclusion

In conclusion, FDG PET reveals abnormalities in relative regional brain glucose metabolism in *Pink1−/−* rats in brain regions that are important to cognition, vocalization, and limb motor control that are also impacted by Parkinson disease. This method may be useful for mechanistic studies of behavioral deficits and therapeutic interventions in translational studies in the *Pink1−/−* PD model.

## Data Availability

The original contributions presented in the study are included in the article/Supplementary material, further inquiries can be directed to the corresponding author.

## Footnotes

^1^ https://www.nitrc.org/projects/tpm_rat/
