# Supplementary material for: Positron emission tomography neuroimaging of [18F]fluorodeoxyglucose uptake and related behavior in the Pink1−/− rat model of Parkinson disease
Source: Front Neurosci. 2024 Oct 15;18:1451118. doi: 10.3389/fnins.2024.1451118 (PMC11520326; doi:10.3389/fnins.2024.1451118)
Supplement: Supplementary file 1 [file Data_Sheet_1.pdf]

Supplementary Table S1 Overview of call types grouped into simple, frequency modulated (FM), and harmonic call categories based on call classification by Wright et al., 2010 and categorization by Hoffmeister et al., 2024.

**Simple**<br>[0-1 frequency modulations]	**FM (Complex)**<br>[2 or more frequency modulations]	**Harmonic (Complex)**<br>[2+ modulations with harmonic component]
Simple<br>(flat, short, upward ramp downward ramp, inverted U)	FM<br>(trill, complex)	Harmonic<br>(Split)
Simple compound<br>(step up, step-down)	FM Compound<br>(multi-step, flat-trill, composite,<br>trill with jumps)	Harmonic Compound<br>(composite with harmonic component)

Supplementary Table S2. Five-Choice Serial Reaction Time Task (5-CSRTT)

(A) Training Stage (TS)	Stimulus Duration (s)	Inter-Trial Interval (s)	Limited Hold (s)	Criterion to move to next stage	Time:
Acclimation	n/a	n/a	n/a	no leftover pellets	10 minutes
Magazine Training 1	n/a	n/a	n/a	no leftover pellets	10 minutes
Magazine Training 2	n/a	n/a	n/a	no leftover pellets	15 minutes
1	30	2	30	70% accuracy	15 minutes
2	10	2	15	70% accuracy	15 minutes
3	5	3	10	70% accuracy	15 minutes
4	2	5	5	70% accuracy	15 minutes
5	1	5	5	70 % accuracy	15 minutes
Long ITI Challenge	same as standard TS	7.5s	same as standard TS		15 minutes
Short SD Challenge	Next TS	same as standard TS	same as standard TS		15 minutes

(B) Testing Week Schedule:	
Day 1:	Standard
Day 2:	Standard
Day 3:	Long-ITI
Day 4:	Maintenance
Day 5:	Short-SD

A) *Training and Test Stage Parameters*. Stimulus duration refers to the duration of light stimulus in each aperture. Inter-trial interval refers to the duration of time between trials. The limited hold refers to the duration of time where the houselight is turned on as a consequence for an incorrect response. B) Testing period schedule.

Supplementary Table S3. Behavior vs. Brain Tests – Significance of interaction of region and genotype

DV	LC	Thal	Prelimbic	SN	Str	Hypogl.	Solitary	Ambig.	PAG	GP	Pons	SMA	PAC m	CdPu	Cereb.	PMA	PAC p	PVA
Long ITI ΔCorrect (%)	0.299	0.612	0.859	0.623	0.576	0.472	0.526	0.372	0.476	0.368	0.748	0.835	0.363	0.625	0.519	0.634	0.709	0.139
Long ITI ΔIncorrect (%)	0.725	0.981	0.284	0.573	0.380	0.449	0.633	0.692	0.575	0.564	0.600	0.953	0.494	0.452	0.577	0.778	0.696	0.788
Long ITI ΔPremature (%)	0.704	0.032*	0.142	0.276	0.034*	0.676	0.863	0.004**	0.899	0.032*	0.021*	0.121	0.320	0.026*	0.094	0.303	0.313	0.154
Long ITI ΔOmission (%)	0.256	0.915	0.722	0.435	0.879	0.327	0.730	0.215	0.314	0.691	0.987	0.882	0.614	0.907	0.785	0.577	0.802	0.163
Short SD ΔCorrect (%)	0.061	0.581	0.074	0.075	0.160	0.478	0.863	0.672	0.287	0.219	0.035*	0.292	0.185	0.147	0.130	0.435	0.840	0.021*
Short SD ΔIncorrect (%)	0.856	0.894	0.817	0.838	0.465	0.732	0.921	0.857	0.201	0.334	0.866	0.191	0.096	0.493	0.245	0.152	0.526	0.352
Short SD ΔPremature (%)	0.880	0.130	0.138	0.216	0.103	0.742	0.918	0.017*	0.987	0.055	0.187	0.159	0.168	0.092	0.120	0.061	0.675	0.177
Short SD ΔOmission (%)	0.039*	0.423	0.072	0.064	0.270	0.541	0.866	0.656	0.734	0.370	0.017*	0.482	0.440	0.247	0.150	0.597	0.662	0.035*
Forelimb Foot Faults	0.496	0.675	0.780	0.135	0.965	0.972	0.506	0.317	0.395	0.875	0.449	0.408	0.362	0.867	0.999	0.453	0.154	0.626
Hindlimb Foot Faults	0.299	0.602	0.992	0.357	0.858	0.527	0.057	0.553	0.451	0.822	0.632	0.689	0.572	0.832	0.455	0.641	0.172	0.990
Time to Traverse Beam (s)	0.924	0.172	0.344	0.801	0.549	0.348	0.743	0.220	0.197	0.275	0.329	0.707	0.296	0.526	0.912	0.970	0.711	0.284
Time to Traverse Final Third (s)	0.661	0.322	0.326	0.919	0.646	0.136	0.831	0.110	0.461	0.505	0.230	0.694	0.199	0.642	0.657	0.425	0.262	0.328
Forelimb Up Steps	0.447	0.733	0.575	0.453	0.468	0.343	0.821	0.208	0.341	0.958	0.188	0.358	0.072	0.599	0.276	0.151	0.918	0.358
Forelimb Down Steps	0.846	0.858	0.897	0.650	0.916	0.072	0.773	0.811	0.211	0.699	0.398	0.091	0.007*	0.898	0.422	0.135	0.156	0.165
Rears	0.758	0.767	0.937	0.272	0.966	0.598	0.198	0.679	0.915	0.813	0.802	0.381	0.002**	0.954	0.242	0.109	0.010*	0.722
Lands	0.866	0.711	0.947	0.276	0.933	0.809	0.281	0.657	0.998	0.815	0.735	0.256	0.001**	0.996	0.208	0.075	0.010*	0.757
Hindlimb Steps	0.863	0.848	0.862	0.238	0.962	0.720	0.850	0.880	0.275	0.295	0.632	0.089	0.017*	0.951	0.361	0.068	0.096	0.814
Power FM (dB/Hz)	0.410	0.176	0.482	0.896	0.408	0.097	0.050	0.854	0.158	0.839	0.964	0.334	0.180	0.388	0.837	0.672	0.305	0.958
Power Simple (dB/Hz)	0.256	0.304	0.975	0.502	0.495	0.102	0.103	0.816	0.340	0.462	0.780	0.219	0.159	0.503	0.964	0.984	0.328	0.901
Peak Frequency FM (kHz)	0.228	0.792	0.622	0.408	0.885	0.111	0.014*	0.665	0.501	0.748	0.754	0.009*	0.037*	0.876	0.004**	0.001**	0.016*	0.887
Peak Frequency Simple (kHz)	0.04*	0.368	0.641	0.404	0.827	0.061	0.011*	0.433	0.345	0.170	0.381	0.146	0.115	0.834	0.140	0.083	0.049*	0.697
Bandwidth FM (kHz)	0.412	0.769	0.225	0.087	0.512	0.02*	0.065	0.236	0.535	0.734	0.529	0.166	0.285	0.524	0.046*	0.296	0.559	0.404
Bandwidth Simple (kHz)	0.074	0.871	0.265	0.280	0.479	0.068	0.018*	0.436	0.627	0.805	0.261	0.263	0.595	0.502	0.182	0.309	0.377	0.725

LC = locus coeruleus, Thal = thalamus, Prelimbic = prelimbic area, SN = substantia nigra, Str. = striatrum, Hypogl. = hypoglossal nucleus, Solitary = solitary nucleus, Ambig. = nucleus ambiguus, PAG = periacqueductal grey, GP = globus pallidus, SMA = secondary motor area, PAC m = parietal association cortex medial area, CdPu = caudate putamen, Cereb. = cerebellum, PMA = primary motor area, PAC p = parietal association cortex posteria area, PVA = primary visual area.

* p < 0.05, ** p < 0.005

Supplementary Table S4. Behavior vs. Brain Tests – Significance of main effect of region

DV	LC	Thal	Prelimbic	SN	Str	Hypogl.	Solitary	Ambig.	PAG	GP	Pons	SMA	PAC m	CdPu	Cereb.	PMA	PAC p	PVA
Long ITI ΔCorrect (%)	0.313	0.793	0.654	0.524	0.638	0.666	0.622	0.869	0.493	0.950	0.591	0.566	0.161	0.595	0.318	0.465	0.761	0.864
Long ITI ΔIncorrect (%)	0.442	0.091	0.046*	0.688	0.043*	0.887	0.981	0.004**	0.122	0.147	0.461	0.575	0.546	0.043*	0.258	0.656	0.808	0.975
Long ITI ΔPremature (%)	0.706	0.086	0.207	0.730	0.145	0.781	0.630	0.077	0.914	0.085	0.199	0.032*	0.280	0.159	0.009*	0.065	0.152	0.424
Long ITI ΔOmission (%)	0.192	0.841	0.796	0.428	0.810	0.687	0.671	0.215	0.176	0.632	0.813	0.653	0.204	0.856	0.583	0.544	0.789	0.779
Short SD ΔCorrect (%)	0.372	0.846	0.521	0.296	0.454	0.936	0.985	0.826	0.859	0.710	0.500	0.605	0.512	0.449	0.482	0.656	0.703	0.582
Short SD ΔIncorrect (%)	0.511	0.247	0.026*	0.224	0.025*	0.898	0.976	0.102	0.901	0.059	0.04*	0.312	0.167	0.032*	0.074	0.231	0.158	0.017*
Short SD ΔPremature (%)	0.442	0.055	0.097	0.749	0.046*	0.709	0.997	0.231	0.822	0.037*	0.123	0.243	0.258	0.048*	0.038*	0.091	0.797	0.393
Short SD ΔOmission (%)	0.132	0.847	0.857	0.196	0.883	0.790	0.743	0.581	0.863	0.720	0.105	0.845	0.716	0.930	0.768	0.996	0.449	0.657
Forelimb Foot Faults	0.675	0.896	0.534	0.214	0.516	0.943	0.900	0.248	0.924	0.455	0.265	0.869	0.076	0.483	0.523	0.385	0.728	0.398
Hindlimb Foot Faults	0.927	0.092	0.104	0.174	0.141	0.633	0.589	0.047*	0.512	0.215	0.106	0.694	0.065	0.133	0.231	0.406	0.256	0.046*
Time to Traverse Beam (s)	0.454	0.951	0.763	0.715	0.612	0.672	0.357	0.702	0.833	0.370	0.817	0.566	0.013*	0.562	0.295	0.232	0.082	0.682
Time to Traverse Final Third (s)	0.629	0.545	0.741	0.964	0.482	0.839	0.543	0.664	0.821	0.188	0.950	0.717	0.104	0.436	0.357	0.413	0.138	0.579
Forelimb Up Steps	0.824	0.552	0.993	0.244	0.952	0.726	0.721	0.887	0.263	0.626	0.727	0.873	0.521	0.991	0.829	0.871	0.966	0.339
Forelimb Down Steps	0.588	0.430	0.974	0.655	0.930	0.235	0.961	0.313	0.525	0.412	0.494	0.892	0.003**	0.968	0.656	0.472	0.019*	0.632
Rears	0.372	0.352	0.051	0.138	0.063	0.093	0.166	0.134	0.410	0.176	0.232	0.771	0.322	0.063	0.108	0.909	0.831	0.143
Lands	0.502	0.340	0.069	0.109	0.084	0.174	0.303	0.144	0.352	0.213	0.277	0.896	0.238	0.081	0.187	0.998	0.724	0.167
Hindlimb Steps	0.942	0.916	0.391	0.144	0.311	0.941	1.000	0.897	0.899	0.548	0.595	0.668	0.069	0.268	0.978	0.795	0.282	0.738
Power FM (dB/Hz)	0.608	0.246	0.296	0.173	0.385	0.460	0.532	0.854	0.714	0.457	0.330	0.847	0.327	0.330	0.965	0.305	0.797	0.698
Power Simple (dB/Hz)	0.342	0.350	0.360	0.038*	0.809	0.267	0.125	0.772	0.133	0.619	0.787	0.153	0.547	0.736	0.875	0.852	0.746	0.306
Peak Frequency FM (kHz)	0.749	0.354	0.259	0.742	0.227	0.991	0.311	0.772	0.846	0.590	0.643	0.883	0.088	0.246	0.348	0.411	0.635	0.487
Peak Frequency Simple (kHz)	0.437	0.563	0.140	0.987	0.164	0.735	0.345	0.787	0.227	0.512	0.229	0.686	0.102	0.176	0.234	0.539	0.713	0.575
Bandwidth FM (kHz)	0.326	0.352	0.592	0.174	0.720	0.744	0.860	0.408	0.208	0.975	0.356	0.949	0.554	0.781	0.871	1.000	0.080	0.063
Bandwidth Simple (kHz)	0.262	0.930	0.106	0.833	0.427	0.549	0.108	0.253	0.802	0.865	0.248	0.163	0.345	0.471	0.386	0.210	0.346	0.445

LC = locus coeruleus, Thal = thalamus, Prelimbic = prelimbic area, SN = substantia nigra, Str. = striatrum, Hypogl. = hypoglossal nucleus, Solitary = solitary nucleus, Ambig. = nucleus ambiguus, PAG = periacqueductal grey, GP = globus pallidus, SMA = secondary motor area, PAC m = parietal association cortex medial area, CdPu = caudate putamen, Cereb. = cerebellum, PMA = primary motor area, PAC p = parietal association cortex posteria area, PVA = primary visual area.

* p < 0.05, ** p < 0.005

Supplementary Table S5. Data.

Genotype	Rat	Timepoint	Age (months)	Weight (g)	LC SUVr	thalamus SUVr	prelimbic area SUVr	substantia nigra SUVr	striatum SUVr	hypoglossal nucleus SUVr	n. of the solitary tract SUVr	ambiguus n. SUVr	periaqueductal gray SUVr	globus pallidus SUVr	pons SUVr	secondary motor area SUVr	parietal assoc. cortex m. SUVr	caudate putamen SUVr	cerebellum SUVr	primary motor area SUVr	parietal association cortex p. SUVr	primary visual area SUVr
Pink1-/-	1	Baseline	10.44	591	1.103	1.216	1.397	0.963	1.222	1.052	1.087	0.883	1.182	1.207	0.968	1.019	0.938	1.236	0.840	1.012	0.879	0.938
Pink1-/-	1	Final	11.64	550	1.169	1.189	1.149	1.018	1.116	1.163	1.202	0.963	1.186	1.151	1.043	0.924	0.893	1.125	0.994	0.924	0.854	0.882
Pink1-/-	2	Baseline	9.12	557	1.101	1.105	1.071	0.977	1.029	0.997	1.060	0.904	1.167	1.078	1.049	0.989	0.902	1.030	0.977	0.977	0.872	0.932
Pink1-/-	2	Final	10.80	582	1.147	1.126	1.107	1.014	1.074	1.169	1.136	0.979	1.147	1.113	1.051	0.929	0.853	1.075	0.992	0.901	0.844	0.856
Pink1-/-	3	Baseline	10.44	591	1.205	1.238	1.324	0.907	1.201	1.101	1.166	0.859	1.201	1.181	1.034	0.995	0.888	1.217	0.923	0.948	0.867	0.894
Pink1-/-	3	Final	11.64	558	1.235	1.184	1.148	1.000	1.083	1.217	1.215	0.984	1.257	1.130	1.110	0.897	0.851	1.091	1.020	0.906	0.838	0.873
Pink1-/-	4	Baseline	9.12	491	1.199	1.231	1.402	0.907	1.209	1.129	1.157	0.878	1.209	1.167	1.006	1.088	0.974	1.232	0.882	1.062	0.865	0.904
Pink1-/-	4	Final	10.80	461	1.144	1.116	1.131	0.992	1.078	1.173	1.167	1.064	1.161	1.066	1.035	0.983	0.845	1.079	1.001	0.939	0.783	0.865
Pink1-/-	5	Baseline	9.12	470	1.098	1.134	1.118	1.001	1.067	1.094	1.162	0.911	1.171	1.079	1.019	0.991	0.743	1.065	0.974	0.961	0.842	0.861
Pink1-/-	5	Final	10.80	471	1.177	1.222	1.095	1.024	1.125	1.129	1.179	0.939	1.274	1.168	1.032	0.948	0.877	1.129	0.984	0.956	0.857	0.859
Pink1-/-	6	Baseline	9.12	565	1.293	1.132	1.135	0.968	1.061	1.215	1.226	1.041	1.205	1.036	1.086	0.959	0.817	1.060	1.001	0.947	0.821	0.869
Pink1-/-	6	Final	10.80	574	1.269	1.186	1.142	1.005	1.102	1.190	1.250	1.030	1.278	1.124	1.106	0.978	0.858	1.108	0.972	0.965	0.822	0.829
Pink1-/-	7	Baseline	9.24	554	1.068	1.115	1.149	1.010	1.076	1.040	1.100	0.968	1.149	1.058	0.998	1.000	0.897	1.074	0.960	0.983	0.852	0.886
Pink1-/-	7	Final	10.56	565	1.105	1.227	1.464	0.962	1.223	1.118	1.169	0.862	1.239	1.191	0.944	1.013	0.895	1.238	0.882	1.013	0.848	0.897
Pink1-/-	8	Baseline	9.12	494																		
Pink1-/-	8	Final	10.80	509	1.130	1.135	1.090	1.082	1.069	1.058	1.087	0.969	1.161	1.106	1.054	0.980	0.876	1.081	0.960	0.935	0.894	0.899
Pink1-/-	9	Baseline	10.44	565	1.290	1.212	1.210	0.996	1.111	1.133	1.211	0.957	1.305	1.133	1.095	0.891	0.844	1.118	0.995	0.881	0.838	0.859
Pink1-/-	9	Final	11.64	536	1.243	1.212	1.237	0.950	1.137	1.116	1.186	0.857	1.283	1.114	1.061	0.982	0.922	1.153	0.931	0.967	0.851	0.890
Pink1-/-	10	Baseline	10.44	565	1.367	1.195	1.167	0.976	1.130	1.212	1.236	0.908	1.284	1.130	1.139	0.932	0.851	1.145	0.988	0.906	0.877	0.875
Pink1-/-	10	Final	11.64	543	1.307	1.180	1.119	1.013	1.113	1.197	1.189	0.883	1.268	1.138	1.131	0.900	0.880	1.116	1.013	0.907	0.822	0.848
Pink1-/-	11	Baseline	9.24	573	1.006	1.138	1.147	1.103	1.083	0.929	0.972	0.893	1.146	1.082	1.017	0.981	0.953	1.086	0.940	0.981	0.901	0.892
Pink1-/-	11	Final	10.56	503	1.201	1.166	1.340	0.960	1.167	1.145	1.234	0.898	1.207	1.122	0.985	1.021	0.946	1.178	0.918	0.978	0.834	0.937
Pink1-/-	12	Baseline	9.12	461	1.202	1.169	1.156	1.004	1.094	1.133	1.200	0.953	1.188	1.131	1.055	0.959	0.886	1.102	1.024	0.950	0.810	0.880
Pink1-/-	12	Final	10.80	491	1.170	1.189	1.122	1.005	1.089	1.110	1.119	1.029	1.233	1.080	1.045	1.000	0.823	1.091	0.963	0.979	0.822	0.838
WT	13	Baseline	10.44	543	1.109	1.230	1.337	1.007	1.180	1.060	1.089	0.914	1.200	1.191	1.010	1.001	0.833	1.197	0.919	0.978	0.761	0.883
WT	13	Final	12.24	543	1.052	1.231	1.426	1.029	1.232	1.018	1.066	0.880	1.191	1.197	0.968	0.992	0.898	1.244	0.867	0.946	0.791	0.890
WT	14	Baseline	9.00	459																		
WT	14	Final	10.44	449	1.171	1.224	1.376	0.949	1.228	1.099	1.187	0.845	1.228	1.195	0.997	0.980	0.894	1.240	0.902	0.970	0.800	0.925
WT	15	Baseline	9.00	468																		
WT	15	Final	10.44	466	1.268	1.199	1.194	0.974	1.117	1.119	1.236	0.938	1.256	1.116	1.039	0.947	0.884	1.121	0.977	0.952	0.850	0.893
WT	16	Baseline	10.44	468	1.100	1.155	1.327	0.964	1.142	0.995	1.108	0.891	1.182	1.079	0.963	1.064	0.922	1.145	0.921	1.008	0.835	0.943
WT	16	Final	12.24	539	1.141	1.177	1.148	1.027	1.121	1.191	1.167	0.993	1.213	1.096	1.052	0.936	0.823	1.120	0.965	0.944	0.759	0.819
WT	17	Baseline	10.44	598	1.146	1.176	1.395	0.978	1.193	1.013	1.066	0.856	1.188	1.126	0.996	1.129	0.942	1.201	0.795	1.054	0.880	0.868
WT	17	Final	12.24	525	1.184	1.209	1.343	0.971	1.209	1.137	1.189	0.846	1.272	1.192	1.000	0.889	0.864	1.230	1.011	0.904	0.732	0.932
WT	18	Baseline	10.32	468	1.094	1.151	1.280	0.951	1.174	1.154	1.216	0.904	1.181	1.143	0.947	0.997	0.892	1.187	0.960	0.966	0.822	0.872
WT	18	Final	12.24	459																		
WT	19	Baseline	9.00	521																		
WT	19	Final	10.44	513	1.133	1.208	1.356	1.016	1.175	1.110	1.172	0.860	1.162	1.132	0.956	1.017	0.892	1.178	0.897	1.004	0.859	0.855
WT	20	Baseline	9.00	517																		
WT	20	Final	10.44	526	1.148	1.219	1.121	1.033	1.115	1.222	1.234	0.981	1.180	1.126	1.015	0.982	0.879	1.116	0.976	0.969	0.847	0.848
WT	21	Baseline	10.32	517	1.116	1.185	1.205	0.995	1.136	1.005	1.082	0.932	1.195	1.142	1.024	1.011	0.871	1.146	0.958	0.976	0.823	0.866
WT	21	Final	12.24	562	1.205	1.163	1.178	1.009	1.097	1.070	1.143	0.918	1.206	1.103	1.087	0.993	0.870	1.095	1.005	0.955	0.856	0.873
WT	22	Baseline	9.00	533																		
WT	22	Final	10.44	542	1.213	1.210	1.187	1.028	1.123	1.098	1.179	0.928	1.436	1.112	1.054	0.950	0.907	1.130	0.945	0.943	0.802	0.841
WT	23	Baseline	9.00	488																		
WT	23	Final	10.44	496	1.421	1.215	1.102	1.014	1.111	1.124	1.247	0.898	1.299	1.157	1.180	0.891	0.865	1.123	1.025	0.914	0.814	0.822
WT	24	Baseline	10.44	543	1.220	1.300	1.494	1.001	1.301	1.038	1.110	0.811	1.335	1.278	1.000	0.968	0.792	1.324	0.886	0.936	0.718	0.847
WT	24	Final	12.24	530	1.054	1.276	1.477	0.983	1.296	1.014	1.027	0.849	1.187	1.251	0.943	1.005	0.905	1.322	0.841	0.990	0.834	0.929
WT	25	Baseline	10.32	543	1.209	1.170	1.250	0.978	1.129	1.117	1.143	0.923	1.195	1.164	1.078	0.944	0.846	1.141	0.965	0.923	0.864	0.883
WT	25	Final	12.24	592	1.207	1.174	1.205	0.983	1.131	1.101	1.148	0.879	1.229	1.128	1.072	0.952	0.853	1.140	1.001	0.927	0.855	0.865
WT	26	Baseline	9.00	525																		
WT	26	Final	10.44	532																		
WT	27	Baseline	9.00	473																		
WT	27	Final	10.44	477	1.222	1.190	1.176	0.989	1.153	1.116	1.186	0.874	1.194	1.169	1.051	0.966	0.890	1.164	0.964	0.970	0.843	0.893
n	54		43	43	43	43	43	43	43	43	43	43	43	43	43	43	43	43	43	43	43	43

Genotype	Rat	Timepoint	Power simple (dB/Hz)	Power FM (dB/Hz)	Peak frequency simple (kHz)	Peak frequency FM (kHz)	Bandwidth simple (kHz)	Bandwidth FM (kHz)	CFFF	CHFF	Total_Time	Time_in_orange	Forelimb up steps	Forelimb down steps	Hindlimb steps	Rears	Lands	Long ITI DeltaCorrect (%)	Long ITI DeltaIncorrect (%)	Long ITI DeltaOmission (%)	Long ITI DeltaPremature (%)	Short SD DeltaCorrect (%)	Short SD DeltaIncorrect (%)	Short SD DeltaOmission (%)	Short SD DeltaPremature (%)
Pink1-/-	1	Baseline	-98.44	-98.26	55.83	57.62	7.64	10.41	2.2	2	3.6	1.8	19	23	24	18	17	6.89	-3.41	-3.48	58.33	-18.38	17.42	0.96	21.11
Pink1-/-	1	Final	-91.65	-93.24	53.40	64.06	10.69	18.36	2.8	2.8	2.4	1.2	14	16	17	14	13	-1.52	2.21	-0.70	49.42	-31.82	7.58	24.24	15.15
Pink1-/-	2	Baseline	-91.75	-95.28	52.67	58.58	11.56	13.23	1.8	2.8	3.2	1.4	4	16	8	12	12								
Pink1-/-	2	Final	-97.22	-98.20	56.97	63.30	11.41	15.65	1.2	2.6	2.8	1.6	0	15	9	11	11								
Pink1-/-	3	Baseline							2.2	1.4	3.6	1.6	5	21	23	12	12	-2.27	-1.52	3.79	23.48	-15.91	0.76	15.15	-10.61
Pink1-/-	3	Final							1.2	1	2.8	1.4	0	9	15	16	16	-3.03	-3.03	6.06	-13.64	-28.03	1.52	26.52	-15.91
Pink1-/-	4	Baseline	-94.43	-95.98	60.56	66.44	13.74	17.52	1.8	2	3	1.8	5	16	29	19	19								
Pink1-/-	4	Final	-95.31	-96.43	66.83	71.62	11.21	15.56	2.2	3.8	5.6	3.8	3	25	25	8	8								
Pink1-/-	5	Baseline	-98.50	-98.52	54.04	61.35	14.44	18.38	3	2.8	5.6	2.6	8	20	25	15	15								
Pink1-/-	5	Final	-101.61	-97.33	63.76	66.75	9.49	16.90	2.2	2.2	4.6	2.4	7	20	27	18	18								
Pink1-/-	6	Baseline	-98.62	-96.48	46.57	56.31	13.05	19.76	3.4	2.6	5	3	9	26	28	13	13								
Pink1-/-	6	Final	-98.24	-94.65	55.05	55.72	9.74	13.48	4.2	4.6	4	2.4	4	15	18	15	15								
Pink1-/-	7	Baseline	-98.97	-96.94	59.04	65.95	11.17	16.84	2	1.6	2	1.2	18	25	32	21	20	15.65	-6.75	-8.90	14.59	8.83	0.07	-8.90	-5.86
Pink1-/-	7	Final	-92.67	-93.20	59.85	65.87	15.08	19.37	1.2	1.4	1.6	1	6	19	16	10	10	3.81	7.84	-11.65	39.09	-28.75	7.84	20.91	29.79
Pink1-/-	8	Baseline	-97.30	-95.35	51.35	57.27	11.55	18.27	1	2	2	1	3	14	20	18	18								
Pink1-/-	8	Final	-99.81	-96.25	54.10	56.78	11.38	17.11	1.4	1.6	2.2	1.2	3	22	26	11	10								
Pink1-/-	9	Baseline	-100.58	-96.53	65.05	67.85	10.38	16.55	1.2	1.8	2.6	1.2	5	16	16	15	15	2.94	-2.99	0.05	20.57	2.94	-5.26	2.32	-13.52
Pink1-/-	9	Final	-93.61	-92.16	54.91	59.67	10.26	16.53	1.8	2	2.4	1.4	9	17	22	18	18	-10.13	3.42	6.72	31.66	-27.24	4.61	22.63	10.01
Pink1-/-	10	Baseline	-98.27	-97.56	57.43	66.43	10.92	14.69	4.2	3.6	4.6	2.8	3	21	22	12	12	0.76	6.82	-7.58	43.94	12.12	-6.82	-5.30	-3.79
Pink1-/-	10	Final	-91.93	-93.24	56.52	63.12	15.68	25.77	2.4	2.6	3.2	1.8	12	15	20	13	12	5.77	4.49	-10.26	38.23	-8.33	9.85	-1.52	4.55
Pink1-/-	11	Baseline	-100.92	-98.33	48.90	59.77	7.68	10.04	1.8	2.2	1.6	1	3	18	20	16	16	-13.72	6.89	6.84	13.70	-0.09	-2.20	2.29	-9.02
Pink1-/-	11	Final	-96.04	-95.65	56.10	62.21	9.61	13.49	1.4	2.4	1.8	1	3	21	17	17	17	-23.05	6.17	16.88	10.50	-50.35	11.57	38.79	-7.12
Pink1-/-	12	Baseline	-94.20	-96.23	55.84	58.64	11.68	14.00	2	2.8	2	1	14	29	26	11	11								
Pink1-/-	12	Final	-97.12	-95.30	53.64	56.57	14.65	20.71	1.4	2.2	2.8	1.2	3	21	15	8	8								
WT	13	Baseline	-82.03	-83.73	52.20	65.65	12.58	24.37	2.2	1.4	3.6	1.8	12	19	23	14	13								
WT	13	Final	-92.59	-91.15	56.46	66.09	12.11	28.69	2.8	1.4	3.6	2	1	11	8	5	4								
WT	14	Baseline	-97.15		42.50		4.28		1	0.8	4.4	2.2	3	11	8	8	8								
WT	14	Final	-97.70		45.83		4.60		0.2	0	3	1.2	0	7	8	8	8								
WT	15	Baseline	-89.41	-87.45	52.53	50.77	11.13	24.94	1.4	1.4	3.4	1.2	9	11	28	14	14								
WT	15	Final	-90.56	-94.73	51.26	60.17	6.59	14.03	2.6	0.6	6	3.4	11	11	17	10	10								
WT	16	Baseline	-82.56	-83.36	57.92	65.45	13.72	28.24	2.2	1	4	2.2	2	13	9	10	9								
WT	16	Final	-94.81	-93.99	56.39	65.51	12.24	22.89	3.2	3	3.4	1.6	5	37	15	14	14	-22.58	-5.71	28.28	8.96	-42.91	3.36	39.55	15.40
WT	17	Baseline	-82.12	-83.12	61.71	72.21	16.74	29.14	0.2	0.8	2.6	1.4	2	18	15	9	8	-25.47	1.04	24.43	24.34	-14.39	-3.79	18.18	7.58
WT	17	Final	-91.20	-92.37	54.97	59.78	14.98	21.66	0.6	0.2	4.2	2	4	30	26	16	16								
WT	18	Baseline	-84.77	-84.52	59.35	64.58	9.68	14.97	0.4	0.6	2.6	1.4	11	18	21	12	11								
WT	18	Final	-90.22	-92.27	53.69	61.96	9.97	14.72	0.2	0.2	2.8	1.4	5	18	15	14	13								
WT	19	Baseline	-94.18	-94.61	63.96	67.59	16.88	29.15	1	1.2	4.6	2.8	7	28	22	8	8								
WT	19	Final	-93.33	-94.09	65.37	70.74	18.84	24.63	1.2	1	4.8	3.4	1	9	2	4	4								
WT	20	Baseline	-97.39	-98.02	54.16	74.80	8.09	23.22	0.2	0	3.4	1.8	1	12	7	6	6								
WT	20	Final	-94.33		48.59		9.70		0	0.2	2.6	1.2	0	13	6	3	3								
WT	21	Baseline	-79.69	-79.49	55.09	60.08	14.95	20.62	2.6	3	4.2	3	5	10	14	14	13								
WT	21	Final	-93.71	-93.96	52.87	58.15	11.49	16.43	2.4	2.6	4	2.4	6	16	10	10	9	1.21	-3.76	2.55	3.84	-42.37	-3.76	46.13	-7.52
WT	22	Baseline	-94.40	-93.38	64.66	66.85	16.14	26.15	0.4	0.4	3.6	1.2	4	21	16	10	9								
WT	22	Final	-95.79	-92.48	68.81	71.69	12.48	23.72	0.8	0.2	3.4	1.8	3	10	9	8	7								
WT	23	Baseline	-104.41		46.71		4.24		0.8	0.6	4	1.6	3	13	9	8	7								
WT	23	Final	-99.24	-91.34	50.65	69.86	4.56	28.99	0.2	0.2	2.4	1.2	5	14	9	6	6								
WT	24	Baseline	-82.54	-80.22	55.67	62.14	15.49	25.51	1.4	0.8	4.2	2	6	22	21	16	15								
WT	24	Final	-96.00	-94.89	66.80	76.33	11.46	18.04	0.2	1	2.6	1.2	9	9	8	12	11	-24.94	2.51	22.43	7.14	-14.19	7.50	6.69	-4.53
WT	25	Baseline	-75.99	-79.65	58.50	68.66	13.83	22.54	0.6	2.4	2.2	1	16	10	20	11	10	-1.75	-1.64	3.39	13.17	8.85	2.32	-11.18	-1.40
WT	25	Final	-90.03	-93.14	57.16	67.47	13.24	21.84	0.4	3.4	2.4	1.2	12	14	10	10	10	-10.41	-2.92	13.34	8.10	-25.61	-3.13	28.74	-12.93
WT	26	Baseline	-90.63	-95.55	58.42	64.54	16.56	24.96	2.2	0.2	4.4	1.8	11	18	12	11	11								
WT	26	Final	-92.23	-92.42	57.15	65.29	16.32	24.39	1.6	0.4	6.2	3.4	3	14	8	9	9								
WT	27	Baseline	-86.23	-92.97	53.88	59.70	12.28	20.80	1.4	1.8	3.8	2.6	6	11	13	8	7								
WT	27	Final	-91.79	-97.40	54.44	60.88	13.27	18.87	0.4	0.8	3.2	2	2	10	9	4	4								
n	54	54	52	48	52	48	52	48	54	54	54	54	54	54	54	54	54	18	18	18	18	18	18	18	18

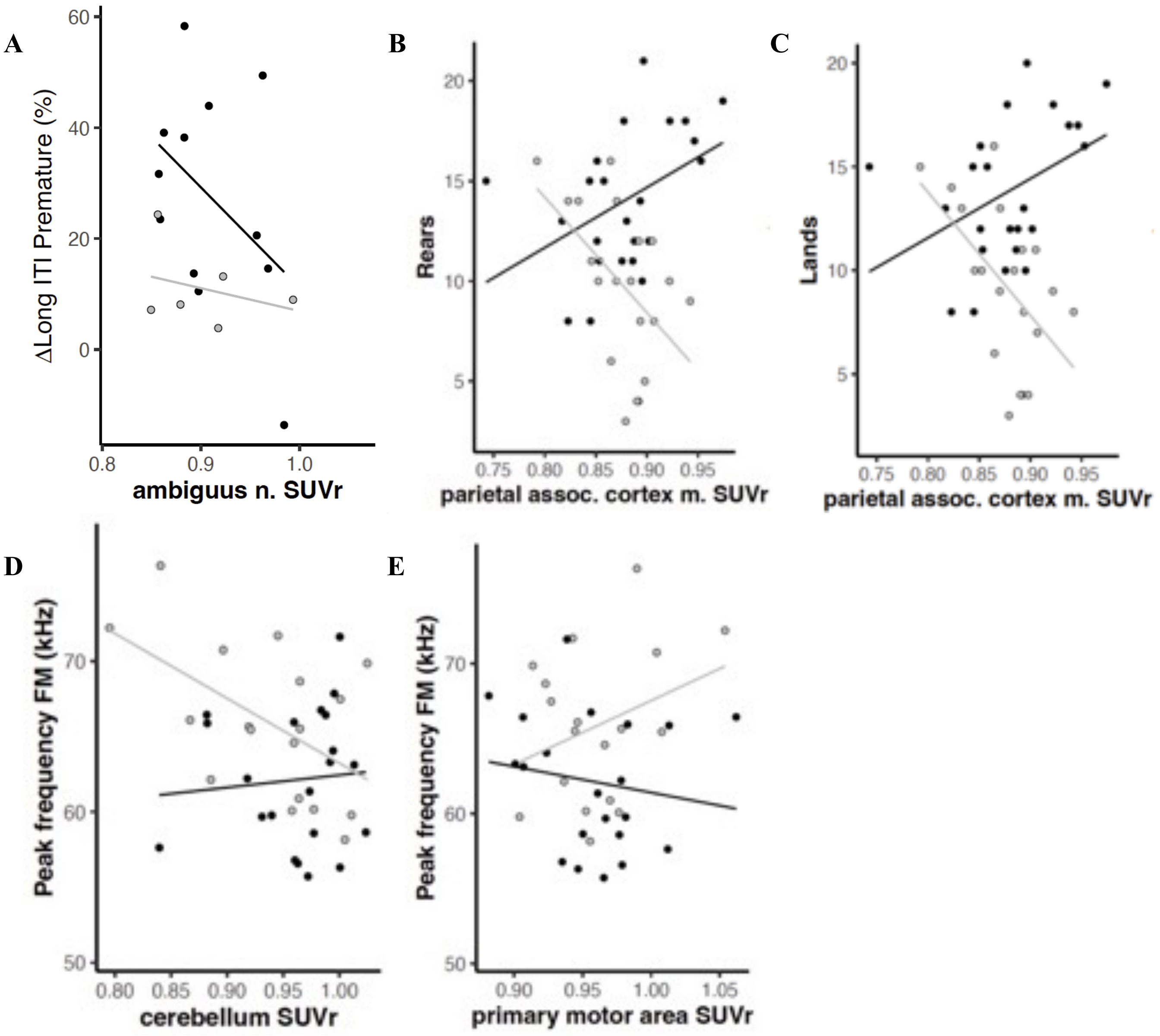

Supplementary Figure S1. Genotype differences in the relationships between behavior and FDG uptake (p<0.005). Significant interactions of region and genotype were observed relating (A) Five-Choice Serial Reaction Time Task long ITI premature responses to nucleus ambiguus, (B) Cylinder Test rears to medial parietal association cortex, (C) Cylinder task lands to medial parietal association cortex, (D) USV peak frequency for FM calls to cerebellum, and (E) USV peak frequency for FM calls to motor cortex. Gray = wildtype rats; black = *Pink1-/-* rats. Lines indicate linear regressions by genotype.

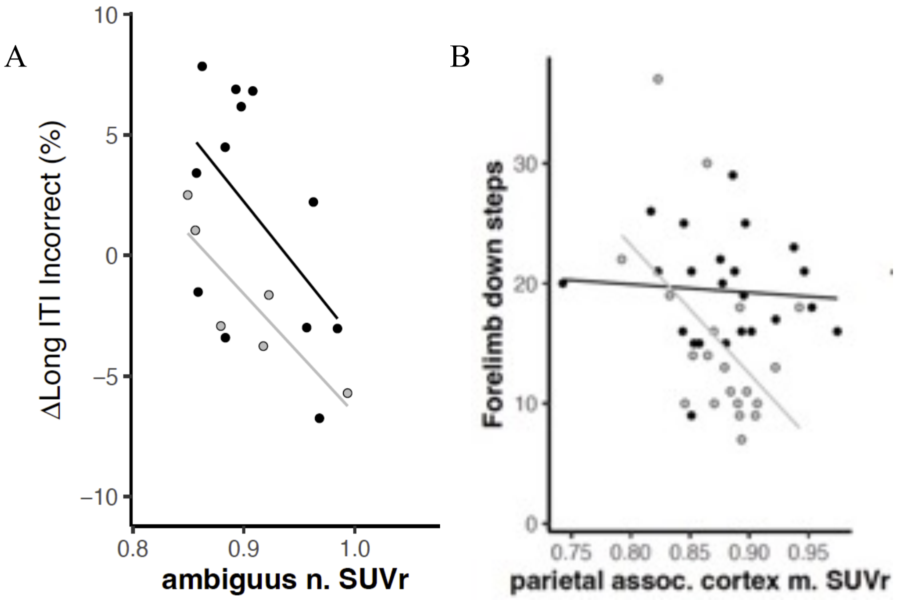

Supplementary Figure S2. Main effects of regional FDG uptake on behavior (p<0.005) relating (A) 5-CSRTT long ITI incorrect responses to nucleus ambiguus and (B) cylinder test forelimb down to medial parietal association cortex. Gray = wildtype rats; black = *Pink1-/-* rats. Lines indicate linear regressions by genotype.
